# Native-like membrane models of *E. coli* polar lipid extract shed light on the importance of lipid composition complexity

**DOI:** 10.1186/s12915-020-00936-8

**Published:** 2021-01-13

**Authors:** Kristyna Pluhackova, Andreas Horner

**Affiliations:** 1 Department of Biosystems Science and Engineering, Eidgenössiche Technische Hochschule (ETH) Zürich, Mattenstr. 26, Basel, 4058 Switzerland; 2 Institute of Biophysics, Johannes Kepler University Linz, Gruberstr. 40, Linz, 4020 Austria

**Keywords:** Molecular dynamics simulations, *E. coli* polar lipid extract, CHARMM36, Martini, Water permeability, Lipid-protein interactions

## Abstract

**Background:**

Lipid-protein interactions stabilize protein oligomers, shape their structure, and modulate their function. Whereas in vitro experiments already account for the functional importance of lipids by using natural lipid extracts, in silico methods lack behind by embedding proteins in single component lipid bilayers. However, to accurately complement in vitro experiments with molecular details at very high spatio-temporal resolution, molecular dynamics simulations have to be performed in natural(-like) lipid environments.

**Results:**

To enable more accurate MD simulations, we have prepared four membrane models of *E. coli* polar lipid extract, a typical model organism, each at all-atom (CHARMM36) and coarse-grained (Martini3) representations. These models contain all main lipid headgroup types of the *E. coli* inner membrane, i.e., phosphatidylethanolamines, phosphatidylglycerols, and cardiolipins, symmetrically distributed between the membrane leaflets. The lipid tail (un)saturation and propanylation stereochemistry represent the bacterial lipid tail composition of *E. coli* grown at 37^∘^C until 3/4 of the log growth phase. The comparison of the *Simple* three lipid component models to the complex 14-lipid component model *Avanti* over a broad range of physiologically relevant temperatures revealed that the balance of lipid tail unsaturation and propanylation in different positions and inclusion of lipid tails of various length maintain realistic values for lipid mobility, membrane area compressibility, lipid ordering, lipid volume and area, and the bilayer thickness. The only *Simple* model that was able to satisfactory reproduce most of the structural properties of the complex *Avanti* model showed worse agreement of the activation energy of basal water permeation with the here performed measurements. The Martini3 models reflect extremely well both experimental and atomistic behavior of the *E. coli* polar lipid extract membranes. Aquaporin-1 embedded in our native(-like) membranes causes partial lipid ordering and membrane thinning in its vicinity. Moreover, aquaporin-1 attracts and temporarily binds negatively charged lipids, mainly cardiolipins, with a distinct cardiolipin binding site in the crevice at the contact site between two monomers, most probably stabilizing the tetrameric protein assembly.

**Conclusions:**

The here prepared and validated membrane models of *E. coli* polar lipids extract revealed that lipid tail complexity, in terms of double bond and cyclopropane location and varying lipid tail length, is key to stabilize membrane properties over a broad temperature range. In addition, they build a solid basis for manifold future simulation studies on more realistic lipid membranes bridging the gap between simulations and experiments.

**Supplementary Information:**

The online version contains supplementary material available at (10.1186/s12915-020-00936-8).

## Background

The picture of a biological membrane as a simple homogeneous slab of lipid molecules that is hydrophobic in the middle and hydrophilic at the surface is obsolete nowadays. Biomembranes are known to be heterogeneous in space and time, to be densely packed with proteins and to comprise hundreds of different lipid species [1]. The biomembrane components are thereby known to influence each other [2–7]. The variation of lipid composition among different membranes, between the two membrane leaflets, in different membrane areas and over time has been recognized as one of very important factors for modulation of membrane properties and thus also the function of membrane-embedded proteins [4].

Thereby, different lipid types play diverse roles. Concentrating on lipid constituents of *E. coli*, the non-lamellar zwitterionic phosphatidylethanolamine (PE) determines the topological organization and functionality of lactose permease LacY [8–10] and is required for cell motility and chemotaxis [11]. In case of the bacterial aquaglyceroporin, GlpF, negatively charged lipids are required for targeting to the cytoplasmic membrane and efficient membrane insertion. However, at concentrations higher than 10%, they hamper the capability of GlpF to transport ribitol [12]. The lipid composition also determines the intrinsic membrane permeability to small solutes [13]. In addition to a large fraction of PE, the bacterial chemoreceptor Tar requires a certain amount of anionic lipids and unsaturated fatty acids for adaptation of correct structure and function [14]. Anionic phospholipids are necessary for SecA-dependent protein translocation across the inner membrane [15], whereby dual recognition of SecYEG and vicinal acidic lipids confers an apparent nanomolar affinity of the motor protein SecA in the absence of ATP ensuring complex stability even during several rounds of ATP hydrolysis [16]. Furthermore, anionic lipids localize the cell division machinery in the middle of the cell [17] and initiate DNA replication [18]. Phosphatidylglycerol (PG) has a significant stabilizing effect on the trimeric ammonia channel AmtB and modulates its activity [19]. Similarly, cardiolipin (CL) stabilizes and functionally regulates the tetrameric water channel aquaporin Z [4]. In addition to functional effects, CL presence in the lipid membrane was related to the ability of oligomer formation for proteins with low oligomeric stability like the sugar transproter SemiSWEET from *Vibrio splendidus*, the Na+/H+ antiporter NhaA from *E. coli*, and the bacterial leucine transporter LeuT from *Aquifex aeolicus* [20]. Furthermore, clusters of strongly charged conic cardiolipin (CL) molecules segregate in areas of membrane curvature and are thus important for processes involving large membrane curvatures like cell division or membrane fusion and fission [21]. Increased CL content additionally ensures survival of bacteria in hyperosmotic solutions [22]. Moreover, in yeast and mitochondria, CL plays essential roles in energy transducing processes by acting as a proton sink and by organization of respiratory complexes [23, 24].

Membrane headgroup composition is organelle and leaflet specific [1], most probably because it influences protein targeting [25], binding strength to the membrane [26], protein oligomerization [20], and functionality of both peripheral and transmembrane proteins [3, 4]. The lipid tail composition on the other hand influences mostly membrane fluidity and thickness [27–30]. It is therefore not surprising that *E. coli* adapts the composition of its lipid tails based on growth conditions, e.g., temperature [31]. Moreover, the presence of cyclopropane moieties in the lipid tails provides *E. coli* membranes resistance against acids [32], heat, and high pressure [33]. Cyclopropanation is a rapid conversion of a double bond into a cyclopropane entity by cyclopropane fatty acid synthase [34]. Recently, lipidomics of *S. aureus* strains resistant or sensitive to specific antibiotics have revealed clear differences in the membrane composition of these bacteria [35]. Interestingly, even the membrane composition of strains resistant to different antibiotics varied [35]. This reveals that bacteria adapt the physicochemical properties of their cell-wall by changing the lipid composition depending on the molecular mode of impact of individual antibiotics. Additionally, enhanced biosynthesis and membrane accumulation of ubiquinone-8 was found to boost bacterial resistance to both hyperosmotic salt shock and permanent salt-induced osmotic stress [36]. Ubiquinone-8 was moreover found to reduce membrane permeability and to improve membrane resistance against deformations [37].

This increased knowledge and awareness of the importance of individual lipids for the function and stability of membrane proteins leads to the use of natural lipid mixtures to mimic the biologically relevant lipid environment in in vitro experiments. In this respect, in vitro experiments are a valuable tool to understand the impact of single lipids on the function and stability of membrane-associated proteins. While in vivo experiments are a valuable approach to study biological processes and single proteins in its full complexity within a living cell, only in vitro systems allow to study and quantify the function of individual membrane proteins in dependence of a wide range of parameters as lipid composition, pH, or different kinds of solutes. The pool of numerous lipid species with a variety of different chain lengths, degrees of saturation, and isomers serves to satisfy the proteins’ needs and create a unique lipid micro-environment in form of a lipid shell around the protein [38, 39]. Moreover, lipids from natural extracts are often cheaper to produce and easier to handle. Therefore, many biophysical investigations are done in natural extracts in vitro, for example in the *E. coli* polar lipid extract (PLE). *E. coli* PLE was found to be essential for proper light-induced transfer of a conformational signal between photoreceptor sensory rhodopsin II, NpSRII, and its cognate transducer, NpHtrII, of *Natronomonas pharaonis* [40] and for studies on effects of antimicrobial peptides on bacterial membranes [41]. It was also used to study the protein translocation by SecA ATPase [42], the bacterial translocon SecYEG opening upon ribosome binding [43], the membrane pore formation by gasdermin D [44], the stiffness map and conformation of interfacial membrane protein moieties with high-speed AFM [45, 46], the determinants of single-file water transport through several aquaporines (AQPs) [47, 48], and KcsA [49] as well as water permeability through hSGTL1 [50]. Another advantage of *E. coli* PLE is its low melting transition temperature of 1−−2^∘^C [51]. This enables experiments over a wide range of temperatures, which in turn allow for the extraction of temperature dependent thermodynamic properties of the system, for example the activation energy of lipid diffusion [52] or of water transport through single-file channels [53].

Molecular dynamics studies are beginning to use lipid compositions similar to that of natural membranes and experimental conditions [54–59]. This development was enabled by both automated preparation of (protein-) membrane systems [60–64] and by extensive parametrization of new lipid species. Already in 2012 Pandit and Klauda have prepared parameters of fatty acids including cyclopropane units and constructed a six-component membrane composition reflecting the PE/PG membrane composition of *E. coli* outside cell poles of bacteria in their stationary stage. The verification of their lipid tail composition was membrane hydrophobic thickness closely matching that of bacterial proteins [55]. Later on, the membrane properties of *E. coli* membranes representing different stages of bacterial growth modeled by different amounts of cy17:0 lipid tails were compared by the same group [29]. In a recent study of a three-component model of a mitochondrial membrane, it was revealed that CL at low concentrations (less than 15%) has only a minor effect on membrane properties [65].

Here, we have prepared complex 14-lipid-component atomistic and coarse-grained (CG) membrane models of *E. coli* PLE reflecting the 3/4 log stage growth, a state used by Avanti$\circledR $ Polar Lipids, Inc. for extraction of *E. coli* PLE. The validation of these models succeeded against our experimental estimations of the activation energy of water permeation as well as against experimental investigations of lipid diffusion and membrane thickness from the literature.

By comparison of the bilayer properties to three simple 3-component membrane models, we show (i) that the diversity of lipid tails, mainly in regard of exact positioning of the unsaturation and cyclopropanation, reduces variations of membrane properties with temperature and (ii) that the complex model better reflects our estimated activation energy of water permeability. The Martini3 CG model perfectly reflects the structural properties of the atomistic model. In addition, the complex lipid model is suitable to house integral membrane proteins as shown for the eukaryotic water facilitator aquaporin-1 (AQP1) and documented by low root mean square deviation (RMSD) values of AQP1 secondary structure relative to the crystal structure. The tetrameric protein causes only a slight increase in lipid ordering and a small reduction of membrane thickness in its vicinity. Although the lipids bind to the surface of AQP1 only temporarily, clear attraction of negatively charged lipids to the cytoplasmic side of the protein could be observed and distinct CL binding sites at the boundary between two protomers unveiled. This finding is so far remarkable as it emphasizes the unique nature of this crevice to bind CL independent of its presence in the biological environment of the human AQP1.

## Results and discussion

*Escherichia coli* adapts to the surrounding conditions by altering the lipid tail composition of its plasma membrane [66]. One possible adaptation mechanism is adding one step to the lipid tail synthesis, thus producing 18:1 ^*cis*11,12^ instead of 16:1 ^*cis*9,10^ single unsaturated lipid tails. If necessary, both unsaturated lipid tails can be converted by cyclopropane fatty acid synthase into lipid tails containing a cyclopropane unit in the position and stereochemistry of the former double bond [34]. Interestingly, the fraction of double bonds and cyclopropane units differs significantly for the two tail lengths, i.e., in *E. coli* PLE membranes grown at 37^∘^C until 3/4 of the log growth phase, two thirds of 16:1 ^*cis*9,10^ lipid tails are converted into cy17:0 ^*cis*9,10^, while only 13% of 18:1 ^*cis*11,12^ were transformed into cy19:0 ^*cis*11,12^ tails [67]. Additionally, CL is known to play important roles for bacterial life, influencing both membrane properties and the functionality of membrane proteins. In previous MD studies of *E. coli* membranes [29, 55] CL was omitted based on the supposition, that most of the *E. coli* plasma membrane is depleted of CL due to its natural overlocalization to the cell poles. However, in liposomes prepared from the *E. coli* PLE, all lipid components are homogenously distributed due to the missing cell-shaping machinery. With the aim to consider all these characteristics of *E. coli* PLE membranes, topologies of 14 different lipids were prepared in both CG (Martini3 force field) and all-atom (CHARMM36 force field) representations. These lipids include 4 CLs, 5 PGs and 5 PEs, all including five different lipid tails: palmitoyl (16:0), palmitoleic acid (16:1 ^*cis*9,10^), cis-11,12-octadecenoic-acid (18:1 ^*cis*11,12^), cis-9,10-methylene-hexadecanoic-acid (cy17:0 ^*cis*9,10^), and cis-11,12-methylene-octadecanoic-acid (cy19:0 ^*cis*11,12^). These tails are denoted as P, Y, V, M, and N, respectively, throughout this work. While in case of the atomistic CHARMM36 force field, it was only necessary to build the topologies of the lipids not present in CHARMM-GUI [60], in case of Martini3 force field the bonded parameters for both cy17:0 and cy19:0 lipid tails compatible with the newest Martini3 lipids, available at the www.cgmartini.nl, had to be newly established. These parameters, collected in the (Additional file 1: Table S4 and Fig. S4), differ from the previously published parameters for lipid tails including cyclopropane units parametrized within the framework of Martini2 [68]. In order to be able to answer the question whether the complexity of the lipid composition of *E. coli* PLE plays a role for its structural or dynamic properties, and in order to shed light on the importance of unsaturation and cyclopropanation position along the lipid tails, three *Simple* membrane models were build and investigated as well. These latter models contain only three lipid types while conserving the proportions of lipid headgroups and lipid tail types (i.e., saturated, including one *cis* double bond or a single cyclopropane unit) of the complex model, but varying the position of the *cis*-bond and cyclopropane in the position 9 and 11. In all models, the lipids are symmetrically distributed in the two leaflets, in order to mimic the in vitro prepared membranes from lipid extracts. We call the 14-lipid membrane composition *Avanti* and the 3-lipid membrane compositions *SimplePOM, SimplePVM*, and *SimplePVJ* throughout this work. Both the force field parameters and the pdb files of the equilibrated membrane structures are available over the website https://github.com/KristynaPluhackova/MD_models_Ecoli-PLE.

### Structural properties of *E. coli* membrane models

**Fig. 1.**
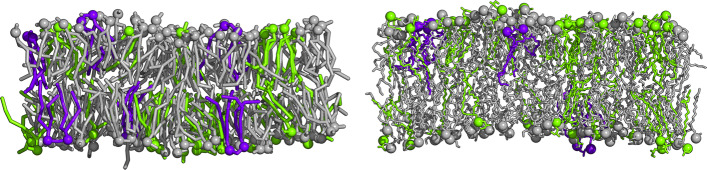
Visualization of simulated systems. Side view of CG (left) and atomistic (right) model of the *Avanti E. coli* PLE bilayers at 310 K. The lipids are colored according to their headgroups, i.e., CLs in purple blue, PGs in green, and PEs in gray. Phosphate atoms or beads are shown as spheres. Water and ions were omitted for clarity. For top views and for top views colored according to lipid tail types, see Additional file 1: Figs. S13 and S14

We have investigated the lipid volume, lipid areas, membrane thickness, lipid ordering, membrane area compressibility, and lipid interleaflet interdigitation of our *SimplePOM, SimplePVM, SimplePVJ*, and *Avanti* membrane models at atomistic and CG resolution in the temperature range between 277 K and 320 K. Exemplarily, the *Avanti* bilayers at 310 K in atomistic and CG resolution are visualized in Fig. 1.

All bilayers follow trends typical for lipid bilayers [27, 69], i.e., the volume per lipid and the area per lipid are increasing whereas the bilayer thickness and lipid tail order are decreasing with increasing temperature. The individual models only differ in the extend and linearity of the respective dependence. The investigated properties are stable over the simulation time as documented by their small standard deviations and as visualized for the case of area per lipid in the Additional file 1: Figs. S5 and S6. The only exceptions are the *SimplePVM* AA simulation at 277 K and *SimplePVJ* AA simulations at 277 and 285 K, which show irregularities in the linear dependance of the structural properties on temperature. The moderate increase of the *trans*-fraction of the lipid tails and decrease in the APL hint to the fact that the lipids partially underwent a transition to a liquid ordered state. However, even those membranes are far from being in a gel state. Visual inspection of the simulated membranes has shown that all other membranes stayed in the liquid-crystalline state. The absence of a liquid-to-gel transition in those simulations is documented by an absence of sudden changes in the area per lipid (shown in the Additional file 1: Figs. S5 and S6) and by a number of other membrane properties analyzed in detail below. Visual investigations of individual simulation snapshots colored either according to their headgroups or according to individual lipid tails (shown in the Additional file 1: Figs. S13 and S14, respectively) revealed a rather homogeneous distribution of both lipid headgroups and tails. The temporary formation of nanodomains and lipid clustering is described below and visualized in the Additional file 1: Figs. S15-S17.

#### Lipid volume

**Fig. 2.**
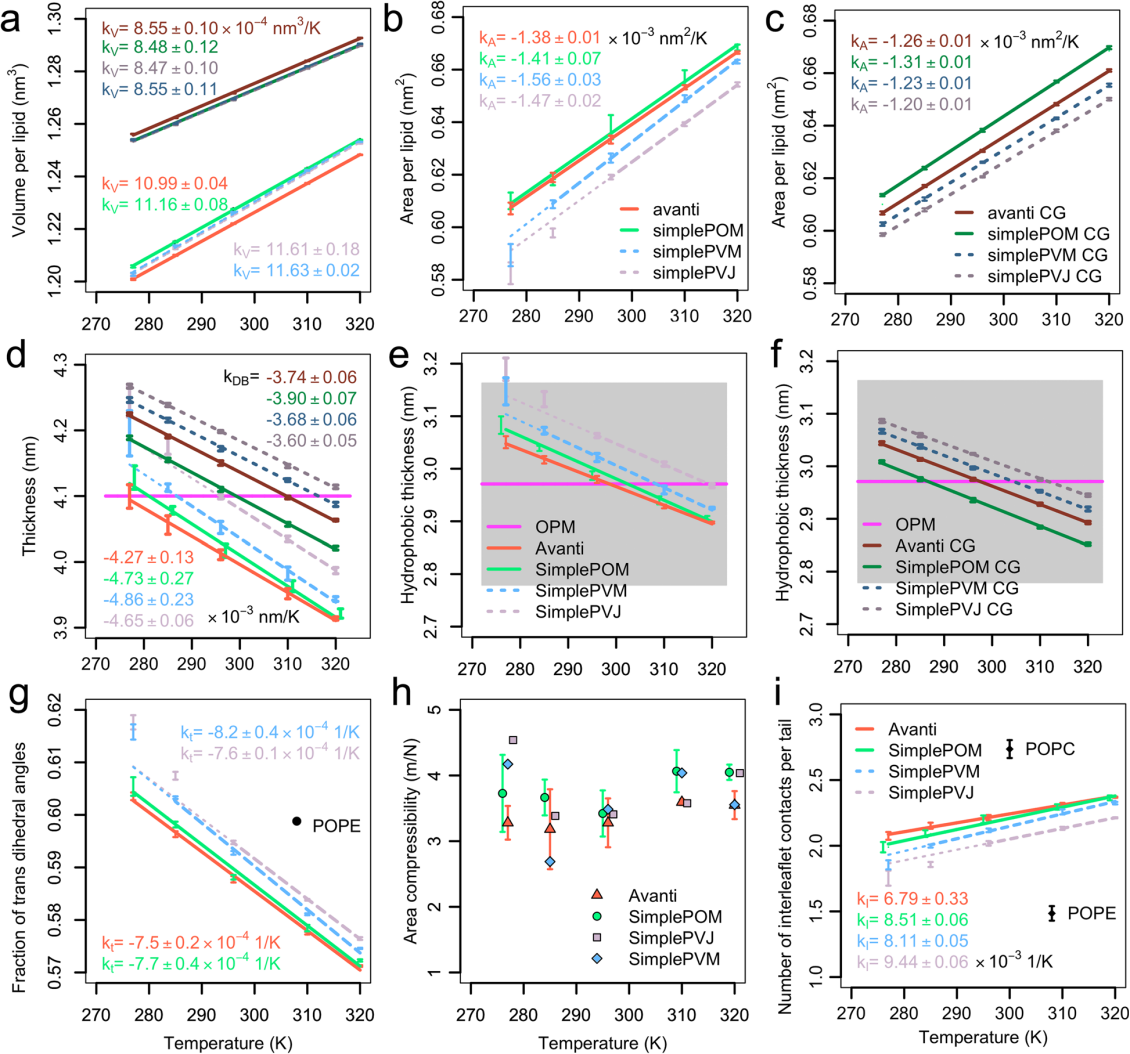
Membrane structural properties. **a** Volumes per lipid, **b** areas per lipid in AA systems, and **c** areas per lipid in CG systems for all models including the volume and area expansivity coefficients. The error bars denote standard deviations over time intervals from all simulations of the given system at the given temperature. The linear fits of the *Avanti* and *SimplePOM* models are shown as full lines, whereas the linear fits of the *SimplePVM* and *SimplePVJ* models are shown as dashed lines. The *SimplePVM* AA APL value at 277 K and the APLs of *SimplePVJ* at 277 K and 285 K were excluded from the fit. The fit extrapolation of the remaining APLs till 277 K is shown as a thin dotted line. **d** Bilayer thickness and hydrophobic membrane thickness at AA (**e**) and CG (**f**) resolution of all investigated systems. For comparison, the experimental bilayer thickness of 4.1 ±0.3 nm [78] as well as the hydrophobic protein thickness of *E. coli* inner membrane proteins 2.97 ±0.19 nm as extracted from the OPM database [79] are shown as magenta lines. The standard error of the hydrophobic protein thickness from the OPM database is visualized as light gray area. Again, the thickness of *SimplePVM* at 277 K and *SimplePVJ* at 277 K and 285 K were excluded from the fit. **g** Fraction of *trans* dihedral angles in lipid tails (C2-C16) from all-atom simulations, the values of *SimplePVM* at 277 K and *SimplePVJ* at 277 K and 285 K were excluded from the fit. **h** Membrane area compressibility for all AA systems. The error bars denote standard errors of the mean of the individual simulations. As only a single repetition simulations were performed for test membrane models *SimplePVM* and *SimplePVJ*, no error bars are given for these systems. The membrane area compressibility values in case of the *SimplePOM* membrane were plotted against temperature values shifted by -1 K, and the values of *SimplePVJ* by 1 K, in order to increase the visibility of the data. **i** Lipid tail interdigitation quantified as the number of interleaflet contacts per tail for all AA systems and for a pure POPE bilayer at 308 K and a pure POPC bilayer at 300 K. The values of *SimplePVM* at 277 K and *SimplePVJ* at 277 K and 285 K were excluded from the fit. The values of the *SimplePOM* membrane were plotted against temperature values shifted by -1 K in order to increase the visibility of the data

The average volumes per lipid (Fig. 2a) increase homogeneously with increasing temperature for all models with high linear fidelity. The atomistic membranes show slightly higher thermal expansivity than the CG models; thereby, the molecular volume of *Avanti* is least temperature dependent of all AA models. Moreover, the CG lipids have by about 4% and 3.5% larger volumes than CHARMM36 lipids in *Avanti* and *Simple* models, respectively. Interestingly, while at AA resolution, the *Avanti* lipids have the smallest molecular volumes, at CG resolution, they are the most voluminous ones. However, the difference at AA resolution amounts to about 0.005 nm^3^ per lipid, corresponding to less than 0.5% of the total volume. The relative difference at CG resolution is even smaller, i.e., less than 0.2% of the total volume. The average molecular volumes of *E. coli* polar lipids are similar to molecular volumes of monounsaturated PGs [70] and PEs [71]. Thereby, PGs have larger molecular volumes then PEs. The temperature dependence of experimental molecular volumes of POPC, POPS, POPG, and POPE is nicely summarized in Figure 6 of Kucerka et al. [30]. As expected, CLs have significantly larger molecular volumes, i.e., the molecular volume of tetraoleoylcardiolipin at 303 K amounts to 2.38 nm^3^ [72]. We conclude that the observed average molecular volumes of our models, consisting of 72% PEs, 23% PGs, and 5% CLs, are as expected. However, it will be interesting to compare those volumes and their temperature dependence with experimentally determined molecular volumes of *E. coli* PLE in the future.

#### Lipid areas

The average areas per lipid (Fig. 2b,c) in all models increase with increasing temperature. Overall, the molecular area expansivities ranging from 0.00120 to 0.00156 *nm*^2^/*K* are similar to those measured for one component lipid bilayer, i.e., mixed saturated and unsaturated lipid chain lipids have *k*_*A*_=0.0014 *nm*^2^/*K* and saturated lipids have *k*_*A*_=0.0019 *nm*^2^/*K* [27]. In the here performed simulations, the average APL of the *Avanti* model is least temperature dependent of all AA models but all over the atomistic models expand slightly faster than the CG models. Control simulations on area expansivity of pure POPC have revealed that this behavior depends on the bilayer type (CHARMM36 area expansivity of POPC amounts to 0.0010 *nm*^2^/*K* [73], while Martini3 POPC area expansivity equals to 0.0013 *nm*^2^/*K*). The thermal area expansivity of the *SimplePOM* atomistic model is only slightly larger than that of the *Avanti* model and the absolute APLs are alike. The APL of the *SimplePVM* lipids is smaller than the APL of the *Avanti* model and this model exhibits the largest thermal expansivity of all AA models. While the thermal area expansivity of the *SimplePVJ* lipids is only slightly higher than of *SimplePOM* model, the absolute APLs of the *SimplePVJ* model are the smallest of all AA models. The APLs at CG resolution depend similarly on the lipid tail composition as the AA models, i.e., the APL of *SimplePVM CG* is smaller than the APL of the *Avanti CG* and the *SimplePVJ CG* has the smallest APL of all CG models. Interestingly, at CG resolution, the *SimplePOM CG* model has significantly larger APL than the *Avanti CG* model, compared to the AA resolution, where the difference is rather marginal. APLs per lipid type calculated by means of the Voronoi tessellation by the tool APL@Voro [74] are shown in Additional file 1: Fig. S7 [74]. As expected based on the APLs of pure lipid bilayers, PEs have the smallest APLs, PGs APLs are by about 10% larger, and CLs have almost twice larger APLs than PEs. Also, the absolute values are similar to the experimental values of pure bilayers. In detail, APLs of PEs, PGs, and CLs in *Avanti* at 310 K ranging from 0.611 to 0.614 nm^2^, 0.673 to 0.695 nm^2^, and 1,165 to 1,212 nm^2^, respectively, are in agreement with the experimental APL of POPE at 308 K of 0.58 nm^2^ [71], APL of POPG at 303 K of 0.661 nm^2^ [70], and APL of tetraoleoylcardiolipin at 303 K of 1.298 nm^2^ [72].

Molecular dynamics simulations as well as experimental investigations have shown that the position of the *cis* double bond in position 9 or 11 dramatically changes the dependence of the area per lipid on the chain length [28], and that the position of the *cis* double bond along the lipid tail strongly effects the bilayer’s phase transition temperature [75, 76]. The lowest phase transition temperatures were obtained for unsaturation in the middle of the lipid tail [76]. Although the differences in the melting temperature of dioctadecenoyl PCs and 1-stearoyl-2-octadecenoyl PCs for unsaturation in position 9 vs 11 amounted only to a few Kelvin [76], the comparison of our *SimplePOM* and *SimplePVM*, which differ only in the position of the *cis* double bond in the oleyol and cis-11,12-octadecenoyl chain, clearly shows that the unsaturation in position 11 reduces the APL and increases the ordering of the lipid tails (compare Fig. 2g and the text below), mainly at low temperatures. This effect is further enhanced if moving the cyclopropane from position 9 to position 11 (*SimplePVM* vs *SimplePVJ* model). The reduced APL and increased ordering lead to irregularities in the linear dependence of the APL on temperature for *SimplePVM* at 277 K and *SimplePVJ* at 277 K and 285 K, hinting to a partial local ordering of the lipid tails. Thus, although *SimplePVM* should be the most alike reduction of the *Avanti* model, the structural properties of this model deviate significantly from the complex *Avanti* model. In order to better mimic the fluidity and higher temperature stability of the *Avanti* model, it is neccessary to substitute the cis-11,12-octadecenoyl chains for oleoyl chains, which are, however, not native to the *E*. *coli* PLE [67].

The here observed ratios of curved and standard APLs, a property indicating membrane curving (shown in the Additional file 1: Fig. S9 [73]), in atomistic resolution are similar to ratios observed for simulation systems of a similar size before [73]. In detail, a pure POPE lipid bilayer simulation using the CHARMM36 force field at 308 K gave APL ratio of 1.040 [73]. The CG models show smaller ratio of curved and standard APLs than the AA models, hinting to more flat membranes at CG resolution.

#### Membrane thicknesses

The membrane thicknesses, estimated locally by GridMAT-MD [77] on a 15 x 15 grid as the distance between the phosphate atoms or PO4 beads are shown in Fig. 2d. They decrease with increasing temperature for all models. The linearity of this decrease is defect only for *SimplePVM* at 277 K and *SimplePVJ* at 277 K and 285 K. The nonlinearity of these systems at those low temperatures matches with increased APL of those systems and their increased lipid tail order. Nevertheless, all values at any temperature lie within the standard error of the experimentally determined membrane thickness of *E. coli* PLE at 298 K of 4.1 ±0.3 nm [78]. The CG models result in about 0.1 nm thicker membranes than the atomistic models. Thereby, the *SimplePOM* lipid CG membrane is by about 0.039 nm thinner than the *Avanti* CG membrane and the *SimplePVM* and *SimplePVJ* CG models are by about 0.024 and 0.047 nm thicker than the *Avanti* CG membrane, respectively. The temperature thickness contractility, ranging from 3.6 ×10^−3^ till 3.9 ×10^−3^ nm/K, is similar for all CG models.

The increased thickness of the *SimplePVM* and *SimplePVJ* models over the *Avanti* model is also present at AA resolution (by 0.05 and 0.1 nm, respectively). However, the *SimplePOM* and the *Avanti* model match better than at the CG resolution. Interestingly, while there is no significant difference in the membrane thickness of atomistic models at higher temperatures (295 K, 310 K, and 320 K) at lower temperatures (285 K and mainly 277 K), the *SimplePOM* lipid bilayer becomes thicker than the *Avanti* membrane (the *p* value of the difference amounts to 3×10^−5^ in both cases). This behavior leads to higher bilayer thickness contractility of the atomistic *SimplePOM* membrane model as compared to the *Avanti* model and to worse linear behavior of the *SimplePOM* membrane thickness with a smaller *R*^2^ value (0.982) of the linear fit. A comparison between all AA models reveals that the thickness of the *SimplePVM* model shows the greatest temperature dependence whereas the *Avanti* model shows the weakest. The membrane thickness contractility values at atomistic resolution ranging from 4.27 ×10^−3^ till 4.86 ×10^−3^ nm/K are similar to those measured for pure monounsaturated PEs [71](POPE k _*DB*_=0.006 nm/K, SOPE k _*DB*_=0.005 nm/K), while the fully saturated DLPE gave a higher thickness contractility of 0.011 nm/K. Monounsaturated PGs give slightly higher (POPG k _*DB*_=0.007 nm/K and SOPG k _*DB*_=0.007 nm/K) and double unsaturated DOPG slightly lower thermal thickness contractility (DOPG 0.003 nm/K) [71].

Additionally, we have estimated the hydrophobic membrane thickness as the transmembrane distance between the boundaries of the polar and hydrophobic lipid parts (Fig. 2e,f). Such hydrophobic thickness can be compared to the hydrophobic protein thicknesses of 171 *E. coli* inner membrane proteins from the Orientations of Proteins in Membranes (OPM) database [79]. The average thickness of the inner membrane proteins amounts to 2.97 ±0.19 nm, with 88% of the proteins having thicknesses between 2.7 and 3.3 nm. The corresponding distribution is shown in the Additional file 1: Fig. S10 [79]. The measured hydrophobic thicknesses of all our models promise good ability of the *E. coli* PLE membrane models to incorporate *E. coli* plasma membrane proteins. Following the trend obtained for the bilayer thickness, the *SimplePOM* CG model is thinner, the *SimplePVM* CG model slightly thicker, and the *SimplePVJ* CG model thicker than the *Avanti* CG model. The hydrophobic thickness of the *Avanti* CG model fits perfectly the average thickness of the transmembrane proteins at 296 K. Also, at AA resolution, the hydrophobic membrane thicknesses of all different models behave similarly to the membrane thicknesses shown in Fig. 2d. The hydrophobic membrane thickness of the *Avanti* lipid composition is most centrally located around the OPM value; however, the maximal difference of 0.02 nm between the *SimplePOM* and *Avanti* models is practically negligible. The *SimplePVM* model is thicker than the *SimplePOM* model and the *SimplePVJ* is the thickest of all models. Additionally, it is expected that the lipid diversity in the *Avanti* composition enables embedding of transmembrane proteins of various hydrophobic thicknesses by segregation of lipids with tail length matching the hydrophobic thickness of the transmembrane protein in its vicinity. Hence, lipid sorting in the protein’s neighborhood can reduce the energy otherwise required for membrane deformation.

#### Lipid ordering

A property directly connected with membrane phase state (gel, liquid crystalline, ordered state) is the amount of dihedral angles in the *trans* state [80, 81]. As expected, the *trans* fraction of lipid tail dihedral angles increases with decreasing temperature (Fig. 2g). The *Avanti* and *SimplePOM* lipid membrane show similar *trans* fractions and temperature dependences. The small difference of 0.15% *trans* fraction of the lipid tails at lower temperatures (285 K and 277 K) cannot explain the above observed decrease in APL of the *SimplePOM* membrane at 285 K and 277 K as compared to the *Avanti* membrane. For comparison, this percentage corresponds to an increase in *trans* fraction of each bilayer upon a temperature decrease of 2 K. The *SimplePVM* model exhibits higher fractions of *trans* dihedral angles at all temperatures with increased temperature dependence as compared to the *SimplePOM* and *Avanti* models. The *SimplePVJ* model is the most ordered one of all. The sudden increase in the fraction of *trans* dihedral angles at 285 K for the *SimplePVJ* model and at 277 K for the *SimplePVM* model is an indicator of partial transition to the liquid-ordered state of the membrane.

For comparison, a *SimplePOM* membrane containing PMPG instead of MMPG lipids and thus containing a higher fraction of saturated lipids was simulated at 277 K for 1.5 *μ*s. This membrane underwent a partial phase transition to a gel state as evaluated by a visual investigation. The average area per lipid between 1 and 1.5 *μ*s of this membrane amounts to 0.54 nm^2^ and the fraction of *trans* dihedral angles is 65.4%. Thus, the here observed values of less than 62% for *SimplePVM* and *SimplePVJ* at 277 K are still rather moderate and far from a lipid membrane in a gel state. For another comparison, the fraction of *trans* dihedral angles of pure POPE bilayer in liquid-crystalline state at 308 K [73] amounts to 59.9%, which is significantly more than for all *E. coli* PLE models studied here at the same temperature.

In case of CG simulations, it is only possible to determine the lipid order parameters of bonds along the lipid tails indicating how well the bonds align with the membrane normal. As expected, the ordering of the CG lipid tails increases with decreasing temperature and is lower in the *SimplePOM* than in the *Avanti* lipid mixture. The corresponding plots can be found in the Additional file 1: Fig. S19.

#### Membrane area compressibility

Unlike other membrane properties such as membrane thickness or area per lipid, the temperature dependence of membrane area compressibility does not follow a single trend. For a single component DPPC bilayer, it was shown that the membrane area compressibility decreases with decreasing temperature, exhibits a minimum of 4.7 m/N at about 12 K above the melting temperature, and then steeply increases upon approaching the melting temperature, reaching a value of 6.3 m/N [82]. This high compressibility close to the melting temperature results from the coexistence of gel and fluid phases [81, 82]. In the gel phase, the membrane becomes rigid with a compressibility value close to 1 m/N [83]. Moreover, membrane rigidity is strongly dependent on the lipid headgroup type [73] and the lipid tail composition, e.g., the amount of cyclopropane units [29, 55].

Figure 2h collects area compressibilities for all atomistic models over the full temperature range spanning from 277 K to 320 K. The compressibility of our *Simple* membranes changes irregularly with increasing temperature, whereas the complex *Avanti* composition exhibits a temperature independent compressibility. Similarly, Pandit and Klauda have observed membrane area compressibility of a simple POPE/POPG system of 4 m/N and of a complex membrane composition titled Top6 of 2.9 m/N at 310 K [55]. The here observed compressibility values lie within the experimental compressibilities obtained at 303 K for POPE (4.29 m/N) and for tetraoleoylcardiolipin (2.92 m/N) [72]. As bacteria house several mechanosensitive proteins, we speculate that the homogeneity of membrane area compressibility of the *Avanti* model over a broad range of physiological temperatures (277–320 K) could be one reason for the high complexity of lipid membrane compositions of native membranes.

Coarse-grained membranes are known to exhibit up to an order of magnitude too low compressibilities [84]. For the sake of completeness, the membrane area compressibility of CG models is included in the Additional file 1: Fig. S18. Values between 1 and 2 m/N match those observed for a pure CG DPPC bilayer in the liquid crystalline state [84]. Coarse-grained bilayers in gel state exhibit membrane area compressibility of about 0.1 m/N [84].

#### Lipid tail interdigitation

Lipid tail interdigitation from one membrane leaflet to the other is speculated to play roles in transleaflet registration of nanodomains [85]. In order to reveal whether the inclusion of lipid tails of different lengths, saturation, and cyclopropanation leads to enhanced lipid tail interdigitation, we have quantified transleaflet contacts as the average number of contacts between lipid tail carbon atoms after exclusion of the terminal methyls between the two leaflets normalized to one lipid tail. The results for AA membrane models is shown in Fig. 2i together with a number of interleaflet contacts per tail for a POPE and a POPC membrane of the same size. The interdigitation of all membranes increases slowly with increasing temperature. The interdigitation of the complex *Avanti* model is the largest, while that of the *SimplePVJ* model the smallest. The latter results likely from the smaller APL and larger lipid tail ordering than in the other *Simple* models. However, the differences are small, and all here studied membranes lie well bellow the interdigitation of a pure POPC bilayer at 300 K. Interdigitation of CG system as well as interdigitation visualized as an overlap of leaflet densities can be found in the Additional file 1: Fig. S12.

### Lipid diffusion

**Fig. 3.**
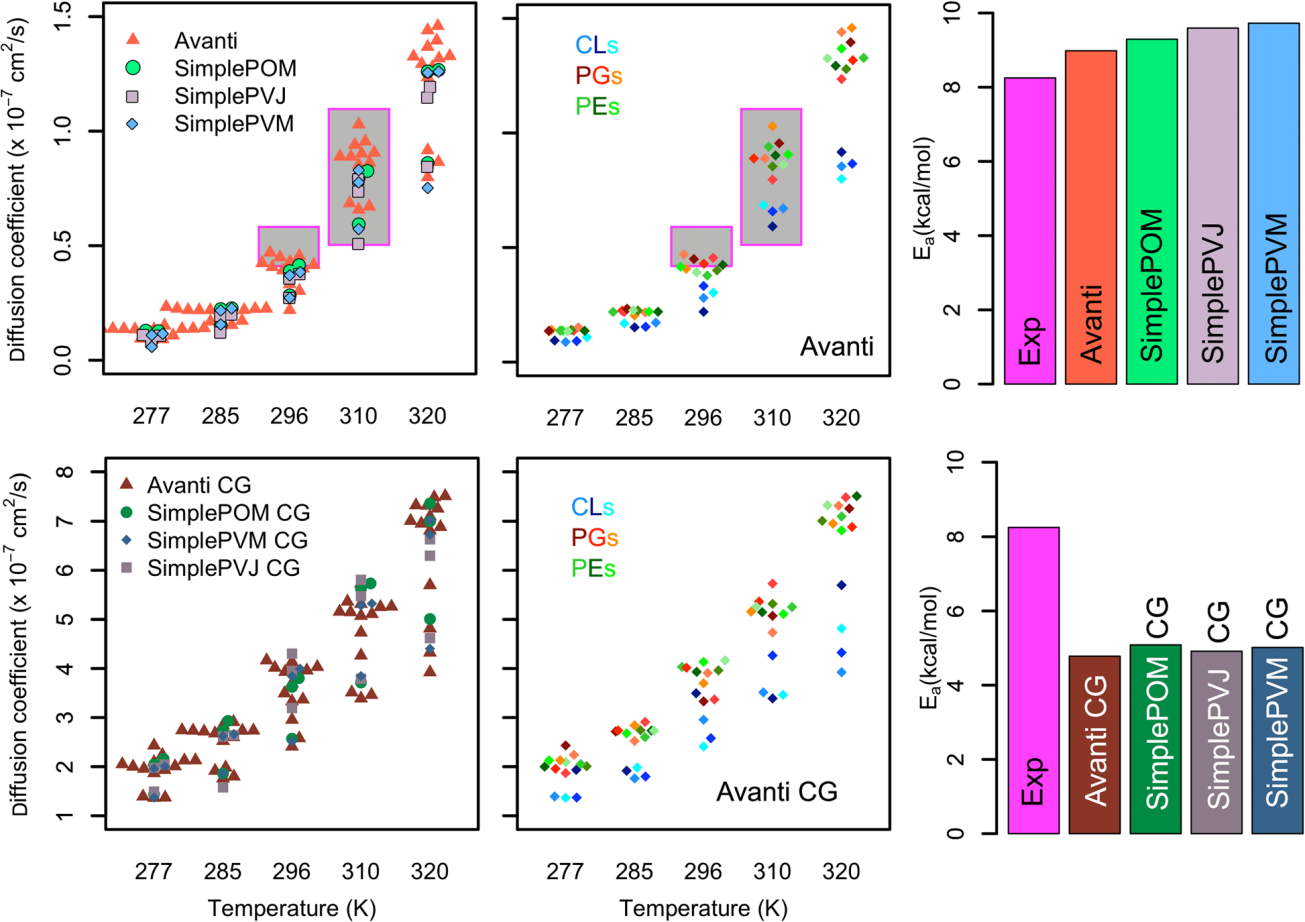
Lipid diffusion. Lipid self-diffusion coefficients at different temperatures for all membrane models (left column) and the average activation energies of lipid self-diffusion (barplot on the right). Results at AA resolution are in the top row and at CG resolution in the bottom row. The magenta-framed gray areas show the span of experimentally determined diffusion coefficients by Jin et al. [88] and Lindlom et al. [52], the magenta bar in the barplots visualizes the experimental activation energy of lipid self-diffusion determined by Lindlom et al. [52]. In the middle, the self-diffusion coefficients of each lipid type in the *Avanti* models are color coded and denoted by a single dot each. For lipid type resolution of the *Simple* models see Additional file 1: Fig. S11. The R’s Beeswarm function [86] was used to create one dimensional scatter plots for each temperature

Figure 3 shows lipid self-diffusion coefficients of all membrane models, at AA and CG resolution, and at different temperatures. Lipid self-diffusion coefficients per lipid type are shown in the Additional file 1: Fig. S11 [86]. As expected the diffusion coefficients increase exponentially with increasing temperature [52]. This exponential dependence allows us to estimate the activation energies (E _*a*_s) of lipid diffusion (Fig. 3) using the Arrhenius equation. At AA resolution, the *Avanti* lipid composition exhibits largest lipid mobility and smallest activation energy of lipid self-diffusion of 8.98 kcal/mol, which is very close to the experimental value of 8.25 kcal/mol obtained by Lindblom et al. for *E. coli* lipids grown at 27^∘^C [52]. The other atomistic models with E _*a*_ of 9.29, 9.59, and 9.72 kcal/mol for *SimplePOM, SimplePVJ*, and *SimplePVM*, respectively, increasingly deviate from the experimental value. The E _*a*_s of the CG models is about half of the atomistic counterparts which likely originates from the different lipid dynamics at the CG resolution. Namely, the friction of the CG lipids is due to the smoother molecular surface significantly lower then of AA lipids. A well-known effect of this reduced friction is 4-5 times larger CG lipid self-diffusion as compared to the atomistic lipids at the same temperature [87]. Otherwise, the dynamics of the CG lipids are similar to the atomistic models, with CL, the heaviest and bulkiest lipids in our membranes, moving the slowest and PGs and PEs having similar mobility.

By comparison of absolute self-diffusion coefficients to the currently available experimental investigations of *E. coli* PLE, two limitations have to be kept in mind. First, both experimental methods, fluorescence recovery after photobleaching [88] and pulsed field gradient NMR spectroscopy [52] measure lipid diffusion on macroscopic scales as compared to the dimensions and timescales reachable by molecular dynamics simulations [89]. Second, the usage of small simulation box size (order of 10 nm) and periodic boundary conditions lead in general to an underestimated diffusion [90]. However, as the CHARMM36 lipid force field was parametrized in comparable box sizes against both structural and dynamic lipid properties [91], the diffusion coefficients obtained from MD simulations of membranes sizes of approximately 10 nm agree well with macroscopic experimental investigations [73, 90]. Therefore, it is not surprising that also the absolute self-diffusion coefficients of our atomistic *E. coli* PLE membrane models compare well to available experimental data. Using fluorescence recovery after photobleaching, Jin et al. estimated lipid self-diffusion coefficients to be 0.4-0.6 ×10^−7^ *cm*^2^/*s* at 296–300 K and 0.5–0.7 ×10^−7^ *cm*^2^/*s* at 307 K [88]. Lindblom et al. measured lipid self-diffusion coefficients of *E. coli* PLE of 0.6 ×10^−7^ *cm*^2^/*s* at 295 K and 1.1 ×10^−7^ *cm*^2^/*s* at 308 K by pulsed field gradient NMR spectroscopy [52].

### Lipid clustering

Membranes of complex lipid composition often show partial lipid separation and formation of nano- or microdomains that can be either stable or strongly fluctuating over time [54]. The formation and stability of those domains depends on many factors like membrane composition, curvature, presence of proteins, DNA, small molecules like ethanol, and coupling to the cytoskeleton, as well as on ion concentration [92, 93]. Such nanodomains play crucial roles in biological processes like transmembrane signaling [94, 95] or transmembrane transport [96]. Molecular dynamics simulations have appeared to perfectly complement experimental investigations on lipid nanodomain formation delivering molecular details and energetics [97]. Due to the high computational efficiency of CG simulations, they are suitable to study long-time processes including phase-separation [98]. Here, we have monitored the homogeneity of lipid bilayers by estimating the number of contacts among lipids carrying different headgroups or lipid tails. Visual investigations of individual simulation snapshots colored either according to their headgroups or according to individual lipid tails (shown in Additional file 1: Figs. S13-S14) revealed a rather homogeneous distribution of lipid headgroups and tails.

The quantitative analysis of relative enrichment and depletion of lipid neighbors and density map estimations (shown in the Additional file 1: Fig S15) revealed that negatively charged lipid headgroups (CL and PG) prefer to interact with neutral PEs in all simulations. This behavior is more pronounced for the more negatively charged CLs than for PGs. The variation between the individual membrane models is small. The 2D density maps (shown in the Additional file 1: Fig S15c) show this distribution in space. While PE lipids are rather homogenously distributed over the bilayer, CLs and PGs complement each other. Because the 2D density maps of CLs evoke the feeling that there is CL clustering, while there is a clear depletion of CL from its direct neighborhood, clustering of CL was evaluated by the GROMACS tool *gmx clustsize*, which counts two molecules to be in a cluster if at least one atom of a molecule is within a certain distance (0.35 in atomistic and 0.65 nm in CG resolution) to the other molecule. The CL cluster distributions (shown in the Additional file 1: Figs. S16-S17) have revealed that in CG simulations about 27% of CLs are monomers, 19% are dimers and 14% are trimers, and the remaining 40% are in higher order oligomers. Thereby, both intra- and interleaflet clusters are included. At atomistic resolution, 1/3 of CLs are monomeric, another 1/3 forms dimers and trimers, thereby dimers are more common than trimers (18-24% of CLs make dimers, while only 6-8% form trimers), and the last 1/3 are higher order oligomers. The number of oligomers stayed constant over time and the variations between the different models are similar to the variations at different temperatures. The lipid tails form transient domains with a slight enrichment in unsaturated and depletion of saturated and cyclopropanated lipid tails and vice versa (see Additional file 1: Fig. S15c). These domains are most pronounced at the lowest temperature and exhibit dynamics of formation and disappearance on a microsecond timescale.

### Water permeability

**Fig. 4.**
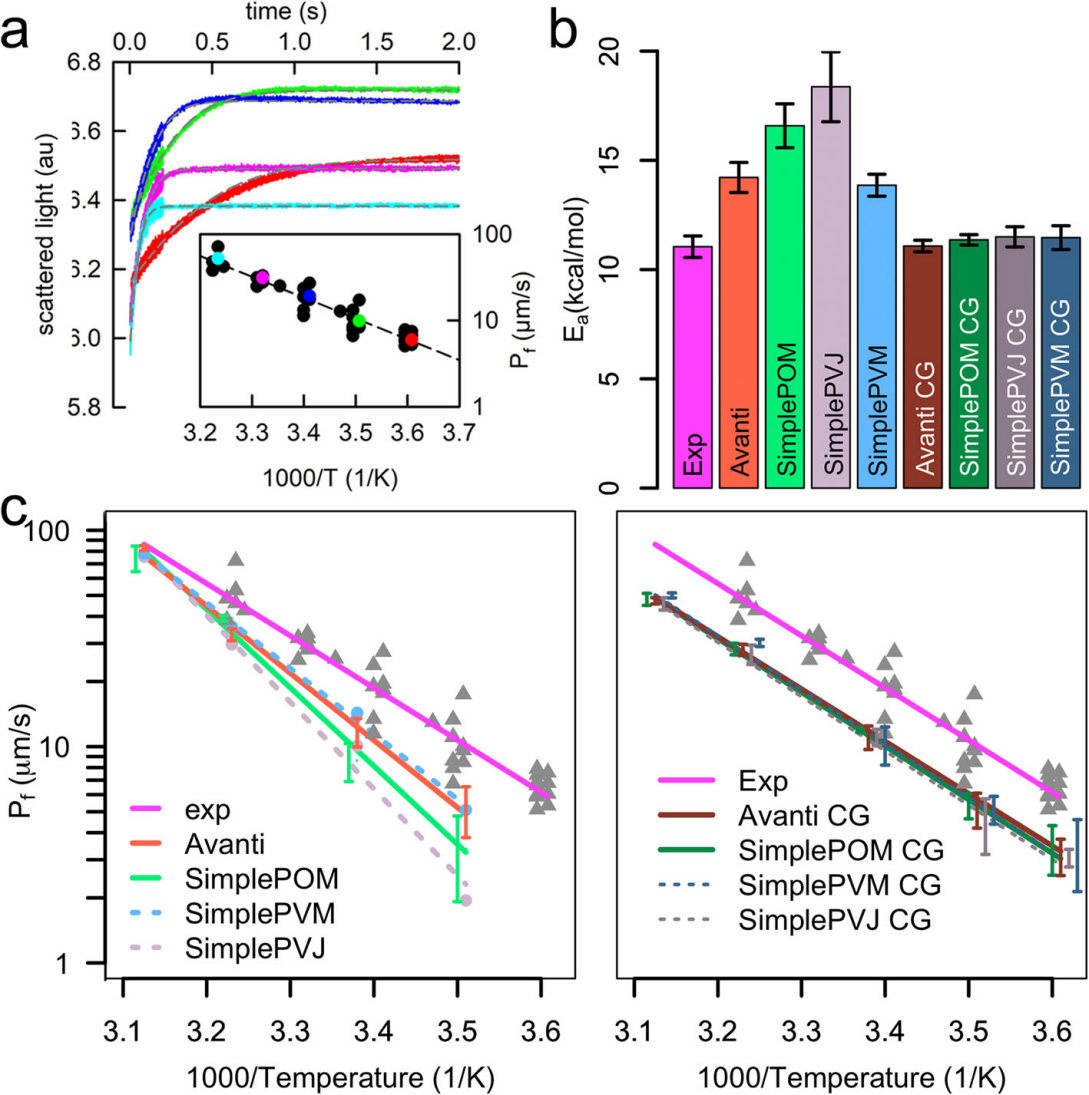
Water permeability over *E. coli* polar lipid extract membranes from in vitro experiment and in silico investigations. **a** Main figure: Representative scattering signals of *E. coli* PLE vesicles (in 100 mM NaCl, 20 mM MOPS at pH 7.40) subjected to an equal amount of hyperosmotic solution (100 mM NaCl, 20 mM MOPS, 150 mM sucrose, pH 7.40) in a stopped-flow apparatus at time zero. The fit of the analytical solution to the data is indicated as dashed gray lines. Different temperatures are color coded. Inset: Arrhenius plot of water flow through *E. coli* PLE membranes. A linear fit to the semilogarithmic plot revealed an E _*a*_ of 11.05 ±0.49 kcal/mol. **b** E _*a*_s of water permeation for all simulated systems and from in vitro experiment, the error bars denote the accuracy of the linear fit performed in **c**. **c** Comparison of water flux over different temperatures from atomistic membranes (left subfigure) and from CG membranes (right subfigure) to in vitro experiment

Although lipid bilayers form a hydrophobic barrier around cells and their organelles, they are not completely water impermeable [13]. Individual water molecules or water pairs pass occasionally the membrane without disturbing the membrane structure, i.e., they do not cause invagination of lipid headgroups and formation of pores. Because basal water permeability is a physiologically important property of native membranes, we have estimated this property and its temperature dependence also experimentally (Fig. 4). As observed for one-component lipid bilayers [73], the basal water permeability increases rapidly with increasing temperature. The large number of temperature values measured and simulated here enabled us to estimate this dependence and to evaluate the activation energy (E _*a*_) of water permeation from the linear fit of the Arrhenius plot, which plots the natural logarithm of the water permeability against the reciprocal temperature (Fig. 4c).

The E _*a*_ of water permeability determined from experiments amounts to 11.05 ± 0.49 kcal/mol. This value is at AA resolution best reproduced by the *Avanti* and by the *SimplePVM* membrane model, i.e., the *Avanti* lipid composition exhibits 14.22 ± 0.69 kcal/mol and the *SimplePVM* model 13.86 ± 0.50 kcal/mol. However, it has to be kept in mind that only single replica simulations have been performed for the *SimplePVM* model leading to a higher uncertainty of the acquired data, even if the error bar in Fig. 4b denoting the error of the fit is smaller. The *SimplePOM* and the *SimplePVJ* model with 16.58 ± 1.00 and 18.37 ± 1.60 kcal/mol, respectively, exhibited higher E _*a*_ of water permeability. The latter suffers again of higher uncertainty. Although two atomistic 1 *μ*s simulations at 277 K per lipid composition were performed, only a few water molecules (7 and 4, and 5 and 9, for *Avanti* and *SimplePOM* lipid composition, respectively) passed the bilayer. This is not surprising given the decreased mobility of both water and lipids and increased membrane thickness and tighter lipid packing at the same time; nevertheless, it lowers the statistical reliability of flux values at low temperatures. Therefore, data from simulations giving less than 1 permeation per 100 ns were excluded from the fit of the Arrhenius plot. All CG membrane models resulted in activation barriers of water permeability in perfect agreement with the experiment. Again, the best was the *Avanti* model with 11.08 ± 0.26 kcal/mol. (The errors denote standard errors of the fit of the Arrhenius plot and all data are summarized in Fig. 4b.) Tables summarizing the total number of water molecules that passed the lipid bilayers and molecular volumes of individual water molecules at given temperatures can be found in the Additional file 1: Tables S5-S6.

Interestingly, although the E _*a*_s of basal water permeation agree well with the experimental value, the absolute flux values in simulations are systematically lower than the experimental ones. As the reason for this discrepancy is not clear, it is advisable to compare relative instead of absolute water flux values between experiment and simulation.

### AQP1 embedded in *E. coli* polar lipid extract

Hydrophobic mismatch between lipids and proteins is an important modulator of protein orientation, aggregation, and functionality [3, 99, 100]. In addition, interfacial lipids serve to stabilize membrane protein oligomers [20] and their selective binding modulates proteins’ structure and function [4, 101]. In order to test how well a transmembrane protein nonnative to *E. coli* is embedded in our *E. coli* PLE *Avanti* model membrane, the human AQP1, one of the best studied tetrameric water channels, was inserted into the membrane and simulated at different temperatures covering a similar temperature range as investigated in pure lipid membranes. AQP1 was shown to functionally reconstitute into large (LUVs) [48] and giant unilamelar vesicles (GUVs) [102] formed of *E. coli* PLE.

**Fig. 5.**
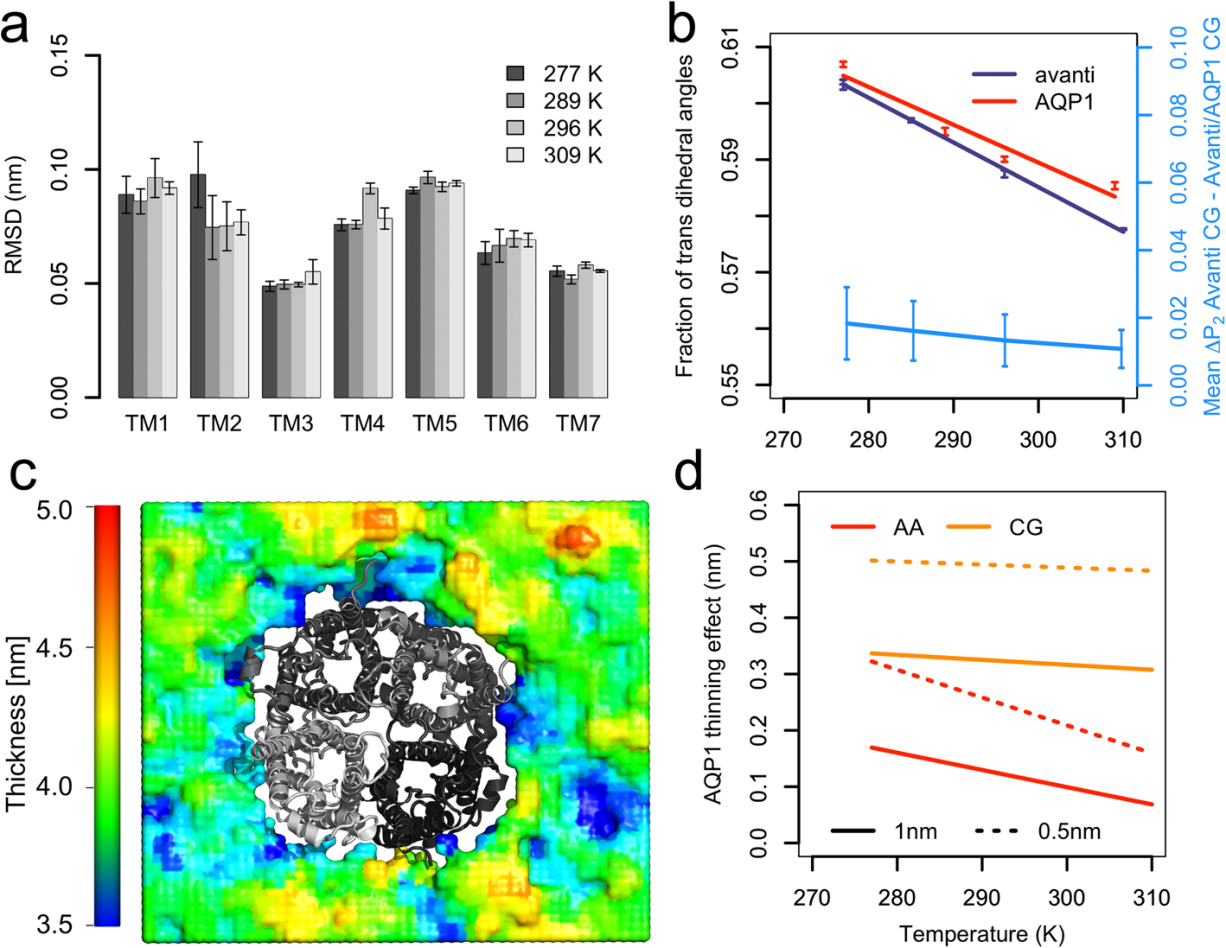
AQP1 secondary structure stability and effects on lipid structural properties. **a** RMSD of individual helices; the error indicates errors of the mean among the individual chains. The functionally important helices TM3 and TM7 form a filter for small charged molecules [103]. **b** Fraction of *trans* dihedral angles of pure *Avanti* bilayer and *Avanti* lipids in the *Avanti*/AQP1 system. In blue, the average difference in the order parameters in the pure *Avanti* CG system and in *Avanti*/AQP1 CG system is shown. The error bars in the latter subplot indicate the standard deviation over individual bonds along the lipid tails. For absolute values of order parameters and for the differences between the *Avanti* CG and *Avanti*/AQP1 CG system for each bond at each temperature, see Additional file 1: Fig. S19. **c** Visualization of local membrane thinning by AQP1 tetramer (shown as gray cartoons) viewed from the cytoplasmic side. **d** Bilayer thinning in 0.5 nm and 1 nm surroundings around AQP1 in both AA and CG resolutions as compared to pure *Avanti* bilayers

The secondary, tertiary, and quaternary structure of the channel are stable over the simulated 500 ns as reflected by the low (less than 0.12 nm) root mean square deviations (RMSDs) of the individual helices shown in Fig. 5a and by RMSDs of the helical bundle in the individual chains and in the whole tetramer. The time evolution of the latter two can be found in the Additional file 1: Figs. S22 and S23.

The protein causes local thinning of the bilayer as documented by Fig. 5b and c. Figure 5b compares membrane thinning within 0.5 nm and 1 nm around the protein in both atomistic and CG resolution. The thinning effect is larger in the thicker CG membrane (average thinning by 0.32 and 0.11 nm for 1 nm radius around CG and AA protein, respectively) and for the closer protein surroundings (average thinning of 0.49 nm and 0.22 nm within 0.5 nm neighborhood of CG and AA AQP1, respectively). In a recent study, the thinning effect of the aquaglycerol channel GlpF in single component bilayers has been estimated to be about 0.6 nm within 1 nm around the protein compared to the bulk thickness [99]. The here observed smaller thinning of the bilayer hints to the fact that our complex *E. coli* PLE membrane model suites well AQP1 hydrophobic thickness. Also, the membrane thickness around the proteins (plotted in the Additional file 1: Fig. S21) reduces only marginally upon heating the system from 277 K to 309 K. In detail, within 0.5 nm around the protein, the membrane thickness decreases by 0.1 nm in CG and by 0.05 nm in AA resolution.

Transmembrane proteins are known to reduce lipid diffusion of vicinal lipids [6, 7, 104]. Here, we have investigated the effect of a transmembrane protein on the *trans* fraction of the lipid tail dihedral angles at AA resolution and the difference in the order parameters of CG lipids, thus investigating the ordering effect of AQP1 on neighboring lipids. Interestingly, the ordering effect in AA resolution is not linear with temperature. As shown in Fig. 5d, the largest increase in the fraction of *trans* dihedral angles takes place at the highest temperature, i.e., at 309/310 K. While the ordering at intermediate temperatures is insignificant, it becomes significant again at the lowest temperature of 277 K. At the CG resolution, the protein causes slight decrease of lipid order parameters thus disordering the lipids. Likely, this disordering results from the local thinning of the bilayer which is more pronounced at the CG than at the AA resolution.

#### Lipid segregation in the vicinity of the protein

**Fig. 6.**
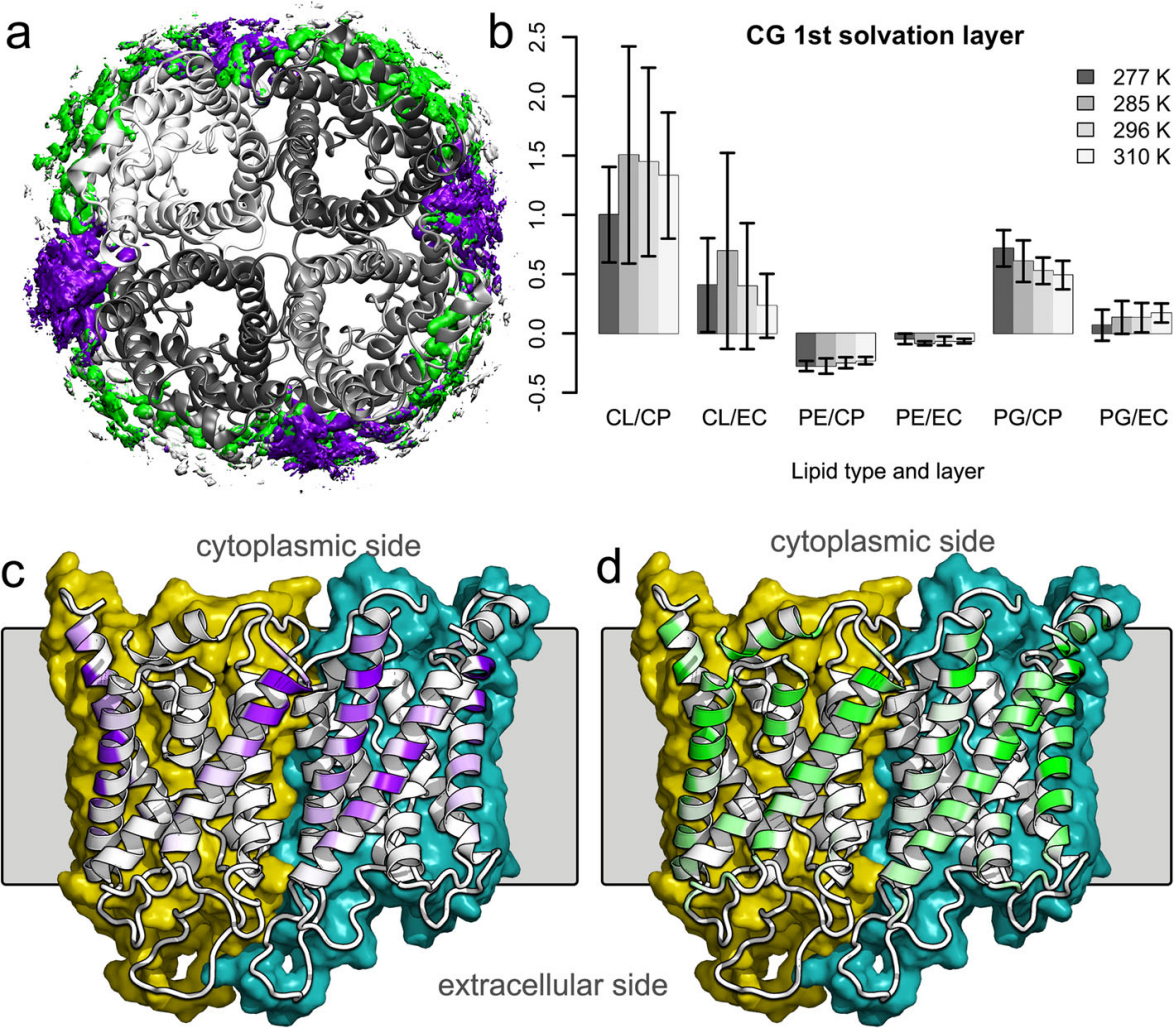
Lipid enrichment around AQP1 in CG simulations. **a** View from the cytoplasmic side on the spatial density of CLs (purple) and PGs (green) around AQP1 tetramer (shown in gray cartoon). **b** Quantification of the lipid enrichment in the first solvation layer (i.e., within 0.65 nm) around AQP1 tetramer relative to the lipid population in the bulk. CL stands for cardiolipins, PG for phosphatidylglycerols, PE for phosphatidylethanolamines, CP for cytoplasmic layer, and EC for extracellular layer. The error bars indicate standard deviations of nine 1 *μ*s intervals from 1 to 10 *μ*s. **c** Cardiolipin binding probability per amino acid (white cartoon—no binding, purple cartoon—high binding) on a tetramer viewed from the side. The membrane is indicated by a gray square; the molecular surface of the individual chains in the front is colored yellow and teal. The cytoplasmic side is at the top and the extracellular on the bottom. **d** PG binding probability per amino acid visualized by a white-green scale (white cartoon denotes no PG binding). The coloring follows otherwise that in **c**

Lipids are increasingly recognized as important allosteric modulators of protein function and structure [105]. Thereby, the effects lipids exert on proteins can result either from specific binding [106] or from changes in general membrane properties like hydrophobic thickness, surface charge, curvature, or surface tension [107, 108]. Also, the function of aquaporines and aquaglyceroporins is influenced by the membrane: the water permeability of AQP4 was shown to depend on membrane properties like membrane area compressibility or thickness [108], and the ribitol transport capability of GlpF is strongly modulated by negatively charged lipids [12]. In case of AqpZ, CL was discovered to bind preferentially to the contact site of monomers in the tetrameric structure [109]. Although AQP1 is an eukaryotic protein spanning the plasma membrane, we have investigated here the binding of *E. coli* polar lipids on its surface. Similarly to AqpZ, CL prefers to bind into the crevice at the contact of two neighboring protomers (see Fig. 6a, c, and d). The rest of the protein surface at the cytoplasmic side of the protein is covered with the negatively charged PGs, which are similarly to CL attracted by a number of positively charged residues (K7, K8, R12, R95, R243, and K245). Accordingly, PEs are depleted in the first solvation layer around AQP1 and negatively charged CLs and PGs enriched at the cytoplasmic side (see Fig. 6, right). The lack of positively charged residues at the extracellular side of the proteins results in a lipid distribution in the first contact layer similar to the composition of the bulk. The time evolutions of lipid enrichment around the protein is shown in the Additional file 1: Fig. S25. These observations are in agreement with enrichment of negatively charged lipids (mainly PIPs) around AQP1 observed by CG MD simulations of AQP1 in a plasma membrane model [110].

Although the spatial density analysis revealed distinct binding sites for CL on AQP1, the analysis of the times each individual lipid spends in contact with this protein (for plots of lipid binding times see Additional file 1: Fig. S24) have revealed a large exchange of lipids on the protein’s surface. In detail, the typical binding times are in the order of hundreds of nanoseconds in CG resolution. The neutral PEs exchange fastest while the negatively charged PGs and CLs stay longer bound on AQP1, mainly at the cytoplasmic side of the protein. The longest binding times are in the order of microseconds and are exhibited by CL and PG at the cytoplasmic side of the protein. The binding characteristics of neutral PEs compares well to a previous study in which CG MD simulations of DPPC binding to 40 different AQPs have shown that there are no tight binding spots of DPPC on AQPs [111]. The maximal observed interaction times of DPPC on AQPs amounted to ∼ 400 ns and the most probable to ∼150 ns [111].

## Conclusions

To enable more accurate MD simulations, we have prepared four model compositions of *E. coli* PLE membranes each of them represented in an atomistic (CHARMM36) and a coarse-grained (Martini3) resolution. Comparing lipid diffusion, membrane thickness, and water permeability to experimental values, the complex *Avanti* composition outperforms the *Simple* three-component compositions. The CG Martini3 model reflected extremely well both the atomistic and the experimental properties of the *E. coli* PLE. Interestingly, although the activation energy of water permeation over the *E. coli* PLE membrane agreed very well between the experiment and the AA and CG simulations of the *Avanti* model, the absolute water flux was consistently underestimated by about 50% in the simulations. Because the reason for this difference is unknown, so far we recommend comparing relative water flux values between experiment and simulation, rather than attempting to compare the absolute values.

Moreover, the comparison of diverse membrane properties over a broad range of temperatures (277–320 K) has revealed important aspects in which the simple three-component bilayers differ from the more realistic 14-component composition, termed *Avanti*. The change with temperature of membrane thickness, area per lipid, and lipid ordering is less steep in the *Avanti* model. Moreover, the membrane area compressibility of the *Avanti* lipid composition remains stable over a broad range of physiological temperatures, i.e., between 277 and 320 K. These results reveal that the complexity in the lipid composition stabilizes membrane properties upon temperature change. Because the functionality of many transmembrane proteins depends on the mechanical properties of the lipid membrane but bacteria are nevertheless exposed to varying temperatures, we hypothesize that one aspect that helps bacteria to survive upon temperature change is the complex lipid composition. Importantly, the variation of the localization of the *cis*-double bond and the cyclopropane moiety between the position 9,10 and 11,12 in the *Simple* models supports our hypothesis. As suggested before, the position of the lipid unsaturation has dramatic effect on the area per lipid [28] and on the phase transition temperature [76]. Our simulations clearly show that unsaturation and cyclopropanation in position 11 reduces the APL and increases the bilayer thickness and the ordering of the lipid tails. Moreover, it decreases lipid mobility, while increasing the activation energy of lipid self-diffusion and water permeation. Thus, although *SimplePVM* should be the most alike reduction of the *Avanti* model, the structural properties of this model deviate significantly from the complex *Avanti* model. On the other hand, the *SimplePVM* model was the only *Simple* model which gave similar activation energy of water permeation to the *Avanti* model and was closest to the experimental value. In order to better mimic the fluidity and higher temperature stability of the *Avanti* model, it is neccesarry to substitute the cis-11,12-octadecenoyl chains for oleoyl chains, as done in the *SimplePOM* model. However, it has to be kept in mind that oleoyl lipid tails are not native to the *E. coli* PLE [67]. Additionally, the agreement of the activation energy of water permeation with experiment is worse in the *SimplePOM* model than in the *SimplePVM* or the *SimplePVJ* model. We therefore encourage usage of the complex *Avanti* model instead of one of the oversimplified *Simple* models and support other scientists by sharing our simulation files via https://github.com/KristynaPluhackova/MD_models_Ecoli-PLE.

Additionally, we have investigated the ability of the *Avanti* models of the *E. coli* PLE to house the eukaryotic AQP1, whose membrane core is structurally conserved with its bacterial homologs. The Martini3 simulations have shed light on lipid binding characteristics to the protein, i.e., the positively charged cytoplasmic side of AQP1 attracts negatively charged lipids, which temporarily bind to the protein in a distinct pattern with CLs intercalating between the monomers and PGs covering the remaining surface of the cytosolic part of the protein. Membrane thinning in the vicinity of the proteins was observed in both AA and CG simulations. At the atomistic resolution, protein’s secondary structure stability was proven over the full range of temperatures. Thus, the *Avanti* models are suitable for future investigations of AQPs in model *E. coli* PLE membranes enabling to study for example protein oligomer formation at CG resolution and water permeability at AA resolution.

## Methods

In order to reveal the importance of lipid diversity and of the localization of each unsaturation and cyclopropanation in positions 9,10 or 11,12 of the lipid tails, four *E. coli* polar lipid extract (PLE) membrane models were built, putting large emphasis on the lipid composition and on their stereochemistry. Because the in vitro used lipid membranes prepared from lipid extracts lack lipid asymmetry between the two lipid leaflets, also the here prepared membrane models contain exactly the same lipids in the upper and lower leaflet. As we intend to reflect the experimentally utilized *E. coli* polar lipid extract, produced by the Avanti$\circledR $ Polar Lipids, Inc., the primary source of information about the lipid headgroup composition and the population of lipid tails for each headgroup type of our membrane models was the product information from the webpage of Avanti$\circledR $ Polar Lipids, Inc.. Each model contains 72% PE, 23% PG and 5% CL, 37% saturated lipid tails, 25% acyl chains with a cyclopropane unit, and 38% unsaturated lipid chains. In the *SimplePOM* lipid composition, all double bonds and cyclopropane moieties are located between carbons 9 and 10 and in the *SimplePVJ* between carbons 11 and 12. In the more realistic *SimplePVM* model, the *cis*-double bond is found in the position 11,12 and the cyclopropane in position 9,10, while in the 14-lipid composition, termed here *Avanti*, double bonds and cyclopropane units of 18-carbon tails are located between carbons 11 and 12, and in case of 16-carbon tails between carbons 9 and 10. Moreover, the stereochemistry of both the cyclopropane units and the *cis* double bonds reflects the natural stereochemistry of *E. coli* membranes [51, 67, 112]. The localization of the double bond was shown to determine the thermodynamics of unsaturated lipids as shown by a different dependence of the areas per lipid of pure lipid bilayers on the chain length [28, 113] and by strong variation of the bilayer melting temperature with unsaturation in different position along the lipid tails [75, 76]. Each phosphate of cardiolipins is carrying a single negative charge, as suggested by the low experimental pKa values of cardiolipins in salt solution including monovalent ions, i.e., Na, K, and Cl [114]. The exact composition is summarized in Table 1 and in the Additional file 1: Table S2. The chemical representations of the molecules, prepared using ChemDraw JS [115], can be found in the Additional file 1: Figs. S1-S3 [115] and a list of all performed simulations is included in the Additional file 1: Table S3.

**Table 1:** Lipid composition of membranes under study

Name	Lipid composition ^*a*^	Tail composition ^*b*^
*SimplePOM*	5% MPPO-CL	39% (P) 16:0
	23% MMPG	37% (O) 18:1 ^*cis*9,10^
	72% POPE	24% (M) cy17:0 ^*cis*9,10^
*SimplePVJ*	5% JPPV-CL	39% (P) 16:0
	23% JJPG	37% (V) 18:1 ^*cis*11,12^
	72% PVPE	24% (J) cy17:0 ^*cis*11,12^
*SimplePVM*	5% MPPV-CL	39% (P) 16:0
	23% MMPG	37% (V) 18:1 ^*cis*11,12^
	72% PVPE	24% (M) cy17:0 ^*cis*19,10^
*Avanti*	1.0% YPMN-CL	
	1.0% YPMV-CL	
	2.1% MPPV-CL	
	0.5% MPPM-CL	
	12.4% PMPG	
	2.1% PNPG	37.4% (P) 16:0
	1.0% YVPG	28.0% (V) 18:1 ^*cis*11,12^
	4.1% PVPG	21.0% (M) cy17:0 ^*cis*9,10^
	2.1% PYPG	9.4% (Y) 16:1 ^*cis*9,10^
	14.5% YVPE	4.2% (N) cy19:0 ^*cis*11,12^
	17.6% PMPE	
	5.7% PNPE	
	8.8% MVPE	
	26.9% PVPE	

^*a*^Molecular fraction of individual lipids; ^*b*^ Percentage of lipid tails, shortcuts are given in brackets. *CL* cardiolipin, *PG* phosphatidylglycerol, *PE* phosphatidylethanolamine, *P* palmitoyl, *Mcis*-9,10-methylene-hexadecanoic-acid, *Ncis*-11,12-methylene-octadecanoic-acid, *Ycis*-11,12-octadecenoic-acid, *V* palmitoleic acid, *O* oleic acid, *Jcis*-11,12-methylene-hexadecanoic-acid. For chemical structure, see Additional file 1: Figs. S1-S3. The proportion of saturated, unsaturated, and cyclopropane-containing tails of both models represents equally well recent experiments [51, 67, 112]

### Parameterisation of lipid tails containing cyclopropane units within the Martini3 framework

Building up on the newest version of the popular CG Martini force field, i.e., Martini3 (obtained from www.cgmartini.nl), we have parametrized phospholipid lipid tails containing cyclopropane units. Martini3 beads including a cyclopropane unit are of the same type as other saturated beads, i.e., C1. For the sake of universality and compatibility, bonds were not reparametrized specifically, but were chosen to be of equal length as in Martini3, i.e., 0.47 nm for all bonds within the hydrophobic lipid tail and 0.51 for the bond connecting the tail to the glycerol. Angle parameters concerning the cyclopropane unit were adjusted against the *E. coli Avanti* membrane simulated at 310 K using the CHARMM36 force field. Comparison of AA and CG angle distributions (shown in Additional file 1: Fig. S4 for both cy17:0 and cy19:0 lipid tails) has revealed that the angle including the cyclopropane bead in the middle has to amount to 125^∘^ with a harmonic constant of 15 kJ/mol/nm^2^. Then, membrane characteristics were compared between atomistic and CG models at all temperature, i.e., 277 K, 285 K, 296 K, 310 K, and 320 K. Detailed parameters for both cy17:0 (M) and cy19:0(N) tails are shown in Additional file 1: Table S4.

### Extension of the lipid topologies using the CHARMM36 force field

The parameters for the cyclopropane units were taken from Pandit et al. [55], who successfully parametrized cy17:0 ^*cis*9,10^ lipid tails and denoted them as M. Here, the lipid topologies for cy19:0 ^*cis*11,12^ tails were prepared and denoted as N. The topologies for the four different CL molecules with mixed lipid tails were prepared, following the standard CHARMM36 topology building strategy, emanating from CL parametrization of Wu et al. [116].

### System preparation and simulation conditions

GROMACS (version 2016.3) molecular dynamics package [117] was used to perform all simulations. The simulation systems, whose lipid compositions are listed in Table 1, were prepared by our established sequential multiscaling methodology [64]. In detail, the membranes at Martini3 CG resolution were prepared using *insane* [63] and solvated by nonpolarizable water (using a single water type W representing 4 atomistic water molecules) and counterions. After energy minimization using 5000 steps of the steepest-descent algorithm, short equilibration simulations with increasing time step were performed to relax the system, i.e., we have performed equilibrations of 200 ps with 2 fs timesteps, 1 ns with 10 fs timesteps, and 1 ns with 20 fs timesteps. The production run simulation of 10 *μ*s was done with 20 fs timesteps. The setup of the simulation conditions and treatment of nonbonded interactions and neighbor lists followed the “new” recommendation of de Jong et al. [118]. The temperature was controlled by the v-rescale thermostat [119] with a time constant of 1 ps for the membrane and the solvent each. The system was semiisotropically pressure coupled every 12 ps to 1 bar in xy- and in z-direction using the Parrinello-Rahman barostat [120] and the compressibility of 3 × 10^−4^ bar ^−1^. The Verlet cutoff scheme was applied with a Verlet-buffer-tolerance of 0.005 kJ/mol/ps. The van der Waals interactions were shifted to zero between 0 and 1.1 nm using the potential-shift-Verlet method; the electrostatic potential was described by the reaction-field method within 1.1 nm using the relative permittivity of the medium equal to 15 [118]. The center-of-mass movement of the system was removed every 100 steps.

The equilibrated CG structures were subsequently converted back to the atomistic resolution using *backward* [121] and the CHARMM36 force field [55, 91, 116]. This force field was chosen after extensive evaluation of different force fields in proper description of both lipid-lipid [73] and lipid-protein interactions [122]. The simulation conditions and treating of nonbonded interactions and neighbor lists followed the tested setup from Pluhackova et al. [73]. In detail, the simulation step at atomistic resolution was 2 fs. The pressure was controlled to be at 1 bar by the Parrinello-Rahman barostat [120] with a time constant of 5 ps. Using a time constant of 0.5 ps, the temperature was kept constant by the Nosé-Hoover thermostat [123, 124]. The center of mass of the system was removed every 100 steps. The Verlet cutoff scheme was used to force-switch the van der Waals interactions to zero in the 0.8–1.2 nm range. The Particle-Mesh-Ewald method [125] was applied to reduce the computational cost of the long-range electrostatic interactions (> 1.2 nm) by evaluating them in the Fourier space at 0.12 nm spacing using a fast Fourier transformation that scales as N.logN. From atomistic simulations performed for 500 ns, the first 100 ns simulations were excluded from the analysis for equilibration purposes. From AA simulations lasting 1000 ns, the first 200 ns were excluded from the analysis. The five temperatures included in our study were 277 K, 285 K, 296 K, 310 K, and 320 K reflecting a temperature just above the melting temperature of *E. coli* PLE [51], an intermediate temperature, room temperature, body temperature, and an elevated temperature well tolerated by *E. coli*, respectively.

The simulation system including AQP1 followed the procedure above. The tetrameric protein was modeled based on the crystal structure 1J4N [126]. Each chain includes residues M1-S249, the free N-terminus carries a positive charge, while the shortened C-terminus is capped by an amine group. All titratable residues are in their standard protonation state at pH 7. Histidines 182 and 211 were chosen to be protonated on *N*^*δ*^ while all other histidines are protonated on *N*^*ε*^. The protonation of His182, a member of the aromatic/arginine constriction side, influences the water permeability of the channel [127]. The protonation of His211 on *N*^*δ*^ provides the opportunity to stabilize the helix 8–helix 7 interaction by building a hydrogen bond with the side chain of Ser202. The atomistic systems with AQP1 were all started from the same CG system, namely after 10 *μ*s at 296 K, keeping the protein position restrained. After the backward conversion to CHARMM36 force field, the minimized crystal tetramer was fitted on the backmapped protein, the overlapping water molecules were removed, and the system was energy minimized in two steps. In the first minimization of 10,000 steps, the protein atoms were kept frozen. In the second minimization procedure of 2000 steps, all atoms were allowed to move freely. The minimization was followed by a 20 ns long simulation with all heavy atoms of the protein position restrained and by a 10 ns simulation with position restrain on the backbone. These equilibration runs as well as the production run simulations were done at 277 K, 289 K, 296 K, and 309 K. The production runs lasted 500 ns each. Additionally, CG AQP1 systems were simulated at 277 K, 285 K, 296 K, and 310 K for 10 *μ*s, each. The secondary structure of each protein chain was separately stabilized using RubberBands as introduced and applied in Wassenaar et al. [61], however, using longer ranged (*r*=1.5 *nm*) but weaker (*F*=50 *kJ*/*mol*/*nm*^2^) bands.

### Analysis

The analysis of basic membrane properties, i.e., lipid area per lipid (APL), curved APL, volume per lipid (VPL), lipid diffusion, membrane thickness, and water permeability, was performed as previously described [73]. If not stated otherwise, the standard deviations of the estimated properties at atomistic resolution were calculated from intervals of all production run simulations at a given temperature. In detail, 100 ns block averages were used at AA resolution after exclusion of initial 100 ns (in case of 1 *μ*s long simulations the initial 200 ns were excluded). Water permeation events were counted over the whole production simulations after exclusion of the initial 100 ns. Structural properties of CG membranes were evaluated over the last microsecond of the simulations and the standard deviations were evaluated from ten 100 ns intervals. Water permeation events at CG resolution were estimated for 1 *μ*s windows after exclusion of the first *μ*s.

In order to be able to compare the hydrophobic membrane thickness to the estimates given in the Orientations of Proteins in Membranes (OPM) database [79], a hydrophobic boundary was defined as the mid distance between the carbonyl carbon and the first methylene carbon in AA representation and glycerol and C1 bead in CG representation for each tail. The hydrophobic thickness amounts to the local distance of these boundaries in the upper and the lower leaflet. The local membrane thickness around AQP1 was calculated on 1 nm sized grid as the difference in the z position of phosphorus atoms found either within 0.5 nm or within 1 nm of the protein in the upper and the lower leaflet.

The lipid tail ordering was estimated by evaluation of the fraction of lipid tail dihedral angles along the tail in *trans* orientation. To this end, the dihedral angles formed by carbons between positions 2 and 16 were evaluated for every single lipid tail and every dihedral angle at every time point of the simulation. Next, all data for individual analysis intervals were combined and the number of *trans* conformations (i.e., dihedral angles from the interval < 130^∘^,230^∘^ > relative to the total number of dihedral angle values in the same time interval) was estimated. Assessment of *trans* fraction of lipid tail dihedral angles was chosen instead of evaluation of order parameters due to its independence of lipid tilt relative to the membrane normal and due to the possibility to estimate a global ordering characteristic independent of the number of different lipids, the presence of double bonds and cyclopropane units, and their localization in the lipid tail.

The order parameters, *P*_2_, of CG lipids were calculated according to

$$P_{2} = \frac {1} {2} \langle 3\cos^{2}\theta - 1 \rangle, $$

where *θ* is the angle between the vector along the bond of interest and the membrane normal. Random orientation is indicated by *P*_2_=0, perfect alignment along the membrane normal by *P*_2_=1 and perfect anti-alignment by *P*_2_=−0.5.

The isothermal membrane area compressibility, *κ*_*T*_, was determined from the fluctuations of the area per lipid using the equation

$$\kappa_{T} = \frac {n_{L}\left(sA_{L}\right)^{2}} {2k_{B}T\langle A_{L} \rangle}, $$

where *k*_*B*_ is the Boltzmann constant, *T* the simulation temperature, 〈*A*_*L*_〉 the average area per lipid, and *sA*_*L*_ the standard error of *A*_*L*_.

Lipid diffusion was estimated individually for each lipid type and temperature on a molecular basis from the Einstein equation as a slope of the lipid mean square displacement versus time (see [73] for more details). The sampling was improved by restarting the calculation every 1 ns. In atomistic resolution, the interval between 10 and 30 ns was used for the fit, while in CG resolution the interval between 100 and 300 ns was utilized.

The activation energies, *E*_*a*_, of water permeability and lipid diffusion were estimated from the Arrhenius plot

$$ln(k) = ln(A) - \frac {E_{a}}{R} \frac{1}{T}, $$

where *k* is the rate constant, *A* the pre-exponential factor, *R* the gas constant, and *T* the absolute temperature in K.

Lipid interdigitation of the two membrane leaflets was previously estimated as the overlap of the density distributions of the two leaflets [128]. In order to enable simple quantification of interdigitation, we suggest here a method in which the number of contacts between lipid tail carbon atoms after exclusion of the terminal methyls (or C4 beads in the CG resolution) between the two leaflets are counted and normalized to one lipid tail.

Lipid clustering was quantified by evaluation of lipid enrichment/depletion in lipid neighborhood. In detail, the number of headgroup contacts within 0.9 nm (i.e., the size of the first solvation shell of CLs’ GL1 bead connecting the two phosphate beads) of each lipid type were calculated using the program *gmx mindist*. As headgroups, the phosphate atoms (or beads in CG resolution) of PG and PE lipids are taken, while for CL, the central carbon of the glycerol connecting the two phosphate units was used (or the corresponding glycerol bead GL1 in CG resolution). For two lipid types, X and Y, the average number of contacts of Y with X per one lipid was estimated in each frame, and then normalized by the average number of contacts of Y lipids to any type of lipid. The plots were generated using R [86, 129], the curved lipid areas were estimated using a home-written code [73] in an idl demo version [130]. Pictures were rendered in PyMOL [131]

### Experimental water permeability of *E. coli* polar lipid extract membranes

Large unilamellar vesicles (LUVs) were prepared from an *E. coli* polar lipid extract (PLE, Avanti Polar Lipids) as described earlier [26]. In brief, PLE was dried on a rotary evaporator, hydrated in a solution containing 100 mM NaCl and 20 mM MOPS buffered at pH 7.4, and put through 21 extrusion cycles using a mini-extruder (Avanti Polar Lipids) stacked with two polycarbonate filters with 100-nm pore sizes. Next, LUVs were subjected to a hyperosmotic solution in a stopped-flow apparatus (SFM-300, Bio-Logic, Claix, France) at several different temperatures ranging from 277 K to 309 K. The intensity of scattered light at a wavelength of 546 nm was monitored at 90^∘^ as described previously (Fig. 4a) [47, 49, 50]. Finally, our recently found analytical solution [48, 132] was used to calculate water permeability values, *P*_*f*_, from light scattering, which in turn served to calculate the activation energy, *E*_*a*_, from the Arrhenius plot for water flow across PLE bilayers (Fig. 4a inset) [133].

## Supplementary Information

**Additional file 1** **Tables S1-S6** and **Figures S1-S25**. **Table S1** - Lipid tail names, shortcuts and stereochemistry. **Table S2** - Molecular composition of each simulation system. **Table S3** - List of performed simulations. **Table S4** - Angle parameters for Martini3 tails including cyclopropane bead. **Table S5** - Details on water permeability at AA resolution. **Table S6** - Details on water permeability at CG resolution. **Fig. S1** - Chemical structures of the lipid headgroups in *E. coli* polar extract membranes. **Fig. S2** - Chemical structures of lipid tails included in this study. **Fig. S3** - ChemDraw representations of lipid molecules in the *Avanti* model. **Fig. S4** - Angle distributions from AA and CG simulations extracted from the *Avanti* model of the *E. coli* polar extract membrane simulations at 310 K. **Fig. S5** - The time evolution of the average area per lipid of AA pure lipid bilayers. **Fig. S6** - The time evolution of the average area per lipid of CG pure lipid bilayers. **Fig. S7** - Area per lipid of each lipid type in pure lipid bilayers at all temperatures and their temperature dependence. **Fig. S8** - Statistical analysis of error estimation of APL. **Fig. S9** - Ratios of the curved area per lipid and the area per lipid estimated form the box size for all systems at all temperatures. **Fig. S10** - Distribution of hydrophobic thickness of 171 transmembrane proteins localized in the inner membrane of *E. coli* obtained from the OPM database. **Fig. S11** - Lipid diffusion in each simulated system for each lipid type including the corresponding activation energies. **Fig. S12** - Interdigitation of membrane leaflets. **Fig. S13** - Lipid mixing at low (277 K) and high (310 K) temperature visualized by coloring lipids according to their headgroups. **Fig. S14** - Lipid tail mixing in individual leaflets at low (277K) and high (310K) temperature visualized by coloring different lipid tail types in different colors. **Fig. S15** - Enrichment and depletion of neighboring lipids. **Fig. S16** - CL clustering at different temperatures in all three membrane models at CG resolution. **Fig. S17** - CL clustering at AA resolution at different temperatures for each lipid mixture. **Fig. S18** - Membrane area compressibility of CG membranes. **Fig. S19** - Order parameters along the lipid tails in CG simulations at different temperatures for all four membrane models and for the *Avanti* model including AQP1. **Fig. S20** - AQP1 embedded in the *E. coli* PLE *Avanti* model at 310 K at AA and CG resolution **Fig. S21** - P-P membrane thickness of *E. coli* PLE *Avanti* model in pure lipid systems and in 0.5 nm and 1 nm surroundings of AQP1 at different temperatures and at AA and CG resolutions. **Fig. S22** - RMSD over time of C _*α*_ atoms within helices of the AA tetramer documenting the stability of the protein’s quaternary structure in AA simulations. **Fig. S23** - RMSD over time of C _*α*_ atoms within helices of the AA monomer documenting the stability of the protein’s tertiary structure in AA simulations. **Fig. S24** - Lipid binding times to CG AQP1 at different temperatures. **Fig. S25** - Time evolutions of lipid enrichment in the first solvation layer around CG AQP1.

## Data Availability

All data generated or analyzed during this study are included in this published article, supplementary information files, or available over the website https://github.com/KristynaPluhackova/MD_models_Ecoli-PLE.

## Abbreviations

AA: All-atom
AQP1: Aquaporin-1
APL: Area per lipid
CG: Coarse-grained
CL: Cardiolipin
CP: Cytoplasmic leaflet
EC: Extracellular leaflet
OPM: Orientations of Proteins in Membranes database
PE: Phosphatidylethanolamine
PG: Phosphatidylglycerol
PLE: Polar lipid extract
RMSD: Root mean square deviation
VPL: Volume per lipid

## References

[1] van Meer G, Voelker DR, Feigenson GW (2008). Membrane lipids: where they are and how they behave. Nat Rev Mol Cell Biol.

[2] Sprong H, van der Sluijs P, van Meer G (2001). How proteins move lipids and lipids move proteins. Nat Rev Mol Cell Biol.

[3] Phillips R, Ursell T, Wiggins P, Sens P (2009). Emerging roles for lipids in shaping membrane-protein function. Nature.

[4] Laganowsky A, Reading E, Allison TM, Ulmschneider MB, Degiacomi MT, Baldwin AJ, Robinson CV (2014). Membrane proteins bind lipids selectively to modulate their structure and function. Nature.

[5] Rabe M, Aisenbrey C, Pluhackova K, de Wert V, Boyle AL, Bruggeman DF, Kirsch SA, Böckmann RA, Kros A, Raap J, Bechinger B (2016). A coiled-coil peptide shaping lipid bilayers upon fusion. Biophys J.

[6] Pluhackova K, Gahbauer S, Kranz F, Wassenaar TA, Böckmann RA (2016). Dynamic cholesterol-conditioned dimerization of the G protein coupled chemokine receptor type 4. PLoS Comp Biol.

[7] Ebersberger L, Schindler T, Kirsch SA, Pluhackova K, Schambony A, Seydel T, Böckmann RA, Unruh T (2020). Lipid dynamics in membranes slowed down by transmembrane proteins. Front Cell Dev Biol.

[8] Chen CC, Wilson TH (1984). The phospholipid requirement for activity of the lactose carrier of Escherichia coli. J Biol Chem.

[9] Bogdanov M, Heacock PN, Dowhan W (2002). A polytopic membrane protein displays a reversible topology dependent on membrane lipid composition. EMBO J.

[10] Dowhan W (2013). A retrospective: use of *Escherichia coli* as a vehicle to study phospholipid synthesis and function. Biochim Biophys Acta.

[11] Shi W, Bogdanov M, Dowhan W, Zusman DR (1993). The pss and psd genes are required for motility and chemotaxis in *Escherichia coli*. J Bacteriol.

[12] Klein N, Hellmann N, Schneider D (2015). Anionic lipids modulate the activity of the aquaglyceroporin GlpF. Biophys J.

[13] Hannesschlaeger C, Horner A, Pohl P (2019). Intrinsic membrane permeability to small molecules. Chem Rev.

[14] Amin DN, Hazelbauer GL (2012). Influence of membrane lipid composition on a transmembrane bacterial chemoreceptor. J Biol Chem.

[15] Kusters R, Dowhan W, de Kruijff B (1991). Negatively charged phospholipids restore prePhoE translocation across phosphatidylglycerol-depleted *Escherichia coli* inner membranes. J Biol Chem.

[16] Winkler K, Karner A, Horner A, Hannesschlaeger C, Knyazev D, Siligan C, Zimmermann M, Kuttner R, Pohl P, Preiner J (2020). Interaction of the motor protein SecA and the bacterial protein translocation channel SecYEG in the absence of ATP. Nanoscale Adv.

[17] Mileykovskaya E, Dowhan W (2005). Role of membrane lipids in bacterial division-site selection. Curr Opin Microbiol.

[18] Sekimizu K, Kornberg A (1988). Cardiolipin activation of dnaA protein, the initiation protein of replication in *Escherichia coli*. J Biol Chem.

[19] Mirandela GD, Tamburrino G, Hoskisson PA, Zachariae U, Javelle A (2019). The lipid environment determines the activity of the escherichia coli ammonium transporter AmtB. FASEB J.

[20] Gupta K, Donlan JAC, Hopper JTS, Uzdavinys P, Landreh M, Struwe WB, Drew D, Baldwin AJ, Stansfeld PJ, Robinson CV (2017). The role of interfacial lipids in stabilizing membrane protein oligomers. Nature.

[21] Mileykovskaya E, Dowhan W (2009). Cardiolipin membrane domains in prokaryotes and eukaryotes. Biochim Biophys Acta Biomembr.

[22] Romantsov T, Guan Z, Wood JM (2009). Cardiolipin and the osmotic stress responses of bacteria. Biochim Biophys Acta Biomembr.

[23] Mileykovskaya E, Penczek PA, Fang J, Mallampalli VKPS, Sparagna GC, Dowhan W (2012). Arrangement of the respiratory chain complexes in Saccharomyces cerevisiae supercomplex III2IV2 revealed by single particle cryo-electron microscopy. J Biol Chem.

[24] Zhang M, Mileykovskaya E, Dowhan W (2002). Gluing the respiratory chain together: cardiolipin is required for supercomplex formation in the inner mitochondrial membrane. J Biol Chem.

[25] Lenarčič T, Albert I, Böhm H, Hodnik V, Pirc K, Zavec AB, Podobnik M, Pahovnik D, žagar E, Pruitt R, Greimel P, Yamaji-Hasegawa A, Kobayashi T, Zienkiewicz A, Gömann J, Mortimer JC, Fang L, Mamode-Cassim A, Deleu M, Lins L, Oecking C, Feussner I, Mongrand S, Anderluh G, Nürnberger T (2017). Eudicot plant-specific sphingolipids determine host selectivity of microbial NLP cytolysins. Science.

[26] Horner A, Goetz F, Tampé R, Klussmann E, Pohl P (2012). Mechanism for targeting the A-kinase anchoring protein AKAP18 *δ* to the membrane. J Biol Chem.

[27] Kučerka N, Nieh M-P, Katsaras J (2011). Fluid phase lipid areas and bilayer thicknesses of commonly used phosphatidylcholines as a function of temperature. Biochim Biophys Acta Biomembr.

[28] Kučerka N, Gallová J, Uhríková D, Balgavý P, Bulacu M, Marrink S-J, Katsaras J (2009). Areas of monounsaturated diacylphosphatidylcholines. Biophys J.

[29] Khakbaz P, Klauda JB (2015). Probing the importance of lipid diversity in cell membranes via molecular simulation. Chem Phys Lipids.

[30] Kučerka N, Heberle FA, Pan J, Katsaras J (2015). Structural significance of lipid diversity as studied by small angle neutron and X-ray scattering. Membranes.

[31] Morein S, Andersson A-S, Rilfors L, Lindblom G (1996). Wild-type *Escherichia coli* cells regulate the membrane lipid composition in a window between gel and non-lamellar structures. J Biol Chem.

[32] Chang Y-Y, Cronan JE (1999). Membrane cyclopropane fatty acid content is a major factor in acid resistance of *Escherichia coli*. Mol Microbiol.

[33] Chen YY, Gänzle MG (2016). Influence of cyclopropane fatty acids on heat, high pressure, acid and oxidative resistance in *Escherichia coli*. Int J Food Microbiol.

[34] Hari SB, Grant RA, Sauer RT (2018). Structural and functional analysis of E. coli cyclopropane fatty acid synthase. Structure.

[35] Hewelt-Belka W, Nakonieczna J, Belka M, Bączek T, Namieśnik J, Kot-Wasik A (2016). Untargeted lipidomics reveals differences in the lipid pattern among clinical isolates of *Staphylococcus aureus* resistant and sensitive to antibiotics. J Proteome Res.

[36] Sévin DC, Sauer U (2014). Ubiquinone accumulation improves osmotic-stress tolerance in *Escherichia coli*. Nat Chem Biol.

[37] Eriksson EK, Edwards K, Grad P, Gedda L, Agmo Hernández V (2019). Osmoprotective effect of ubiquinone in lipid vesicles modelling the *E. coli* plasma membrane. Biochim Biophys Acta Biomembr.

[38] Zhang G, Keener JE, Marty MT (2020). Measuring remodeling of the lipid environment surrounding membrane proteins with lipid exchange and native mass spectrometry. Anal Chem.

[39] Teo ACK, Lee SC, Pollock NL, Stroud Z, Hall S, Thakker A, Pitt AR, Dafforn TR, Spickett CM, Roper DI (2019). Analysis of SMALP co-extracted phospholipids shows distinct membrane environments for three classes of bacterial membrane protein. Sci Rep.

[40] Mosslehy W, Voskoboynikova N, Colbasevici A, Ricke A, Klose D, Klare JP, Mulkidjanian AY, Steinhoff H-J (2019). Conformational dynamics of sensory rhodopsin ii in nanolipoprotein and styrene–maleic acid lipid particles. Photochem Photobiol.

[41] Berry T, Dutta D, Chen R, Leong A, Wang H, Donald WA, Parviz M, Cornell B, Willcox M, Kumar N, Cranfield CG (2018). Lipid membrane interactions of the cationic antimicrobial peptide chimeras melimine and cys-melimine. Langmuir.

[42] Catipovic MA, Bauer BW, Loparo JJ, Rapoport TA (2019). Protein translocation by the SecA ATPase occurs by a power-stroke mechanism. EMBO J.

[43] Knyazev DG, Lents A, Krause E, Ollinger N, Siligan C, Papinski D, Winter L, Horner A, Pohl P (2013). The bacterial translocon SecYEG opens upon ribosome binding. J Biol Chem.

[44] Mulvihill E, Sborgi L, Mari SA, Pfreundschuh M, Hiller S, Müller DJ (2018). Mechanism of membrane pore formation by human gasdermin-D. EMBO J.

[45] Karner A, Nimmervoll B, Plochberger B, Klotzsch E, Horner A, Knyazev DG, Kuttner R, Winkler K, Winter L, Siligan C, Ollinger N, Pohl P, Preiner J (2016). Tuning membrane protein mobility by confinement into nanodomains. Nat Nanotechnol.

[46] Preiner J, Horner A, Karner A, Ollinger N, Siligan C, Pohl P, Hinterdorfer P (2015). High-speed AFM images of thermal motion provide stiffness map of interfacial membrane protein moieties. Nano Lett.

[47] Horner A, Siligan C, Cornean A, Pohl P (2018). Positively charged residues at the channel mouth boost single-file water flow. Faraday Discuss.

[48] Horner A, Zocher F, Preiner J, Ollinger N, Siligan C, Akimov SA, Pohl P (2015). The mobility of single-file water molecules is governed by the number of H-bonds they may form with channel-lining residues. Sci Adv.

[49] Hoomann T, Jahnke N, Horner A, Keller S, Pohl P (2013). Filter gate closure inhibits ion but not water transport through potassium channels. Proc Natl Acad Sci U S A.

[50] Erokhova L, Horner A, Ollinger N, Siligan C, Pohl P (2016). The sodium glucose cotransporter SGLT1 is an extremely efficient facilitator of passive water transport. J Biol Chem.

[51] White GF, Racher KI, Lipski A, Hallett FR, Wood JM (2000). Physical properties of liposomes and proteoliposomes prepared from *Escherichia coli* polar lipids. Biochim Biophys Acta Biomembr.

[52] Lindblom G, Orädd G, Rilfors L, Morein S (2002). Regulation of lipid composition in Acholeplasma laidlawii and *Escherichia coli* membranes: NMR studies of lipid lateral diffusion at different growth temperatures. Biochemistry.

[53] Horner A, Pohl P (2018). Single-file transport of water through membrane channels. Faraday Discuss.

[54] Ingólfsson HI, Melo MN, van Eerden FJ, Arnarez C, Lopez CA, Wassenaar TA, Periole X, de Vries AH, Tieleman DP, Marrink SJ (2014). Lipid organization of the plasma membrane. J Am Chem Soc.

[55] Pandit KR, Klauda JB (2012). Membrane models of E. coli containing cyclic moieties in the aliphatic lipid chain. Biochim Biophys Acta Biomembr.

[56] Enkavi G, Javanainen M, Kulig W, Róg T, Vattulainen I (2019). Multiscale simulations of biological membranes: the challenge to understand biological phenomena in a living substance. Chem Rev.

[57] Monje-Galvan V, Klauda JB (2015). Modeling yeast organelle membranes and how lipid diversity influences bilayer properties. Biochemistry.

[58] Gocheva G, Ivanova N, Iliev S, Petrova J, Madjarova G, Ivanova A (2020). Characteristics of a folate receptor- *α* anchored into a multilipid bilayer obtained from atomistic molecular dynamics simulations. J Chem Theory Comput.

[59] Marrink SJ, Corradi V, Souza PCT, Ingólfsson HI, Tieleman DP, Sansom MSP (2019). Computational modeling of realistic cell membranes. Chem Rev.

[60] Jo S, Kim T, Iyer VG, Im W (2008). CHARMM-GUI: a web-based graphical user interface for CHARMM. J Comput Chem.

[61] Wassenaar TA, Pluhackova K, Moussatova A, Sengupta D, Marrink SJ, Tieleman DP, Böckmann RA (2015). High-throughput simulations of dimer and trimer assembly of membrane proteins. The DAFT approach. J Chem Theory Comput.

[62] Pluhackova K, Wassenaar TA, Kirsch S, Böckmann RA (2015). Spontaneous adsorption of coiled-coil model peptides K and E to a mixed lipid bilayer. J Phys Chem B.

[63] Wassenaar TA, Ingolfsson HI, Böckmann RA, Tieleman DP, Marrink SJ (2015). Computational lipidomics with *insane*: a versatile tool for generating custom membranes for molecular simulations. J Chem Theory Comput.

[64] Pluhackova K, Wassenaar TA, Böckmann RA, Rapaport D, Herrmann JM (2013). Molecular dynamics simulations of membrane proteins. Membrane Biogenesis.

[65] Wilson BA, Ramanathan A, Lopez CF (2019). Cardiolipin-dependent properties of model mitochondrial membranes from molecular simulations. Biophys J.

[66] Siliakus MF, van der Oost J, kengen SWM (2017). Adaptations of archaeal and bacterial membranes to variations in temperature, pH and pressure. Extremophiles.

[67] Stuart LJ, Buck JP, Tremblay AE, Buist PH (2006). Configurational analysis of cyclopropyl fatty acids isolated from *Escherichia coli*. Org Lett.

[68] Groenewald W, Bulacu M, Croft A, Marrink S-J. Molecular dynamics of mycolic acid monolayers; 2019. 10.26434/chemrxiv.7881215.v1. preprint.

[69] Zhuang X, Makover JR, Im W, Klauda JB (2014). A systematic molecular dynamics simulation study of temperature dependent bilayer structural properties. Biochim Biophys Acta Biomembr.

[70] Pan J, Heberle FA, Tristram-Nagle S, Szymanski M, Koepfinger M, Katsaras J, Kučerka N (2012). Molecular structures of fluid phase phosphatidylglycerol bilayers as determined by small angle neutron and X-ray scattering. Biochim Biophys Acta Biomembr.

[71] Kučerka N, van Oosten B, Pan J, Heberle FA, Harroun TA, Katsaras J (2015). Molecular structures of fluid phosphatidylethanolamine bilayers obtained from simulation-to-experiment comparisons and experimental scattering density profiles. J Phys Chem B.

[72] Pan J, Cheng X, Sharp M, Ho C-S, Khadka N, Katsaras J (2015). Structural and mechanical properties of cardiolipin lipid bilayers determined using neutron spin echo, small angle neutron and X-ray scattering, and molecular dynamics simulations. Soft Matter.

[73] Pluhackova K, Kirsch SA, Han J, Sun L, Jiang Z, Unruh T, Böckmann RA (2016). A critical comparison of biomembrane force fields: structure and dynamics of model DMPC, POPC, and POPE bilayers. J Phys Chem B.

[74] Lukat G, Krüger J, Sommer B (2013). APL@ Voro: a Voronoi-based membrane analysis tool for GROMACS trajectories. J Chem Inf Model.

[75] Huang C (2001). Mixed-chain phospholipids: structures and chain-melting behavior. Lipids.

[76] Lewis RNAH, McElhaney RN, Yeagle PL (2005). The mesomorphic phase behavior of lipid bilayers. The structure of biological membranes.

[77] Allen WJ, Lemkul JA, Bevan DR (2009). GridMAT-MD: a grid-based membrane analysis tool for use with molecular dynamics. J Comput Chem.

[78] Lind TK, Wacklin H, Schiller J, Moulin M, Haertlein M, Pomorski TG, Cárdenas M (2015). Formation and characterization of supported lipid bilayers composed of hydrogenated and deuterated *Escherichia coli* lipids. PloS ONE.

[79] Lomize MA, Lomize AL, Pogozheva ID, Mosberg HI (2006). OPM: orientations of proteins in membranes database. Bioinformatics.

[80] Siu SWI, Pluhackova K, Böckmann RA (2012). Optimization of the OPLS-AA force field for long hydrocarbons. J Chem Theory Comput.

[81] Sun L, Böckmann RA (2018). Membrane phase transition during heating and cooling: molecular insight into reversible melting. Eur Biophys J.

[82] Kirsch SA, Böckmann RA (2019). Coupling of membrane nanodomain formation and enhanced electroporation near phase transition. Biophys J.

[83] Rand RP, Parsegian VA (1989). Hydration forces between phospholipid bilayers. Biochim Biophys Acta Biomembr.

[84] Wang Y, Gkeka P, Fuchs JE, Liedl KR, Cournia Z (2016). DPPC-cholesterol phase diagram using coarse-grained molecular dynamics simulations. Biochim Biophys Acta Biomembr.

[85] Skotland T, Sandvig K (2019). The role of PS 18:0/18:1 in membrane function. Nat Commun.

[86] R Core Team (2017). R: a language and environment for statistical computing.

[87] Marrink SJ, Risselada HJ, Yefimov S, Tieleman DP, de Vries AH (2007). The MARTINI force field: coarse grained model for biomolecular simulations. J Phys Chem B.

[88] Jin AJ, Edidin M, Nossal R, Gershfeld NL (1999). A singular state of membrane lipids at cell growth temperatures. Biochemistry.

[89] Lautner L, Pluhackova K, Barth NKH, Seydel T, Lohstroh W, Böckmann RA, Unruh T (2017). Dynamic processes in biological membrane mimics revealed by quasielastic neutron scattering. Chem Phys Lipids.

[90] Venable RM, Ingólfsson HI, Lerner MG, Perrin BS, Camley BA, Marrink SJ, Brown FLH, Pastor RW (2017). Lipid and peptide diffusion in bilayers: the Saffman–Delbrück model and periodic boundary conditions. J Phys Chem B.

[91] Klauda JB, Venable RM, Freites JA, O’Connor JW, Tobias DJ, Mondragon-Ramirez C, Vorobyov I, MacKerell AD, Pastor RW (2010). Update of the CHARMM all-atom additive force field for lipids: validation on six lipid types. J Phys Chem B.

[92] Schmid F (2017). Physical mechanisms of micro- and nanodomain formation in multicomponent lipid membranes. Biochim Biophys Acta Biomembr.

[93] Cebecauer M, Amaro M, Jurkiewicz P, Sarmento MJ, Šachl R, Cwiklik L, Hof M (2018). Membrane lipid nanodomains. Chem Rev.

[94] Schmidt C, Kim D, Ippolito GC, Naqvi HR, Probst L, Mathur S, Rosas-Acosta G, Wilson VG, Oldham AL, Poenie M, Webb CF, Tucker PW (2009). Signalling of the BCR is regulated by a lipid rafts-localised transcription factor, Bright. EMBO J.

[95] Gahbauer S, Böckmann RA (2016). Membrane-mediated oligomerization of G protein coupled receptors and its implications for GPCR function. Front Physiol.

[96] Weinrich M, Worcester DL, Bezrukov SM (2017). Lipid nanodomains change ion channel function. Nanoscale.

[97] Bennett WFD, Tieleman DP (2013). Computer simulations of lipid membrane domains. Biochim Biophys Acta Biomembr.

[98] Carpenter TS, López CA, Neale C, Montour C, Ingólfsson HI, Di Natale F, Lightstone FC, Gnanakaran S (2018). Capturing phase behavior of ternary lipid mixtures with a refined martini coarse-grained force field. J Chem Theory Comput.

[99] Jensen MØ, Mouritsen OG (2004). Lipids do influence protein function—the hydrophobic matching hypothesis revisited. Biochim Biophys Acta Biomembr.

[100] Killian JA (1998). Hydrophobic mismatch between proteins and lipids in membranes. Biochim Biophys Acta Biomembr.

[101] Gahbauer S, Pluhackova K, Böckmann RA (2018). Closely related, yet unique: distinct homo-and heterodimerization patterns of G protein coupled chemokine receptors and their fine-tuning by cholesterol. PLoS Comp Biol.

[102] Boytsov D, Hannesschlaeger C, Horner A, Siligan C, Pohl P (2020). Micropipette aspiration-based assessment of single channel water permeability. Biotechnol J.

[103] de Groot BL, Grubmüller H (2001). Water permeation across biological membranes: mechanism and dynamics of aquaporin-1 and GlpF. Science.

[104] Niemelä PS, Miettinen MS, Monticelli L, Hammaren H, Bjelkmar P, Murtola T, Lindahl E, Vattulainen I (2010). Membrane proteins diffuse as dynamic complexes with lipids. J Am Chem Soc.

[105] Contreras F-X, Ernst AM, Wieland F, Brügger B (2011). Specificity of intramembrane protein–lipid interactions. Cold Spring Harb Perspect Biol.

[106] Dawaliby R, Trubbia C, Delporte C, Masureel M, Van Antwerpen P, Kobilka BK, Govaerts C (2015). Allosteric regulation of G protein–coupled receptor activity by phospholipids. Nat Chem Biol.

[107] Lee AG (2004). How lipids affect the activities of integral membrane proteins. Biochim Biophys Acta Biomembr.

[108] Tong J, Briggs MM, McIntosh TJ (2012). Water permeability of Aquaporin-4 channel depends on bilayer composition, thickness, and elasticity. Biophys J.

[109] Schmidt V, Sidore M, Bechara C, Duneau J-P, Sturgis JN (2019). The lipid environment of *Escherichia coli* Aquaporin Z. Biochim Biophys Acta Biomembr.

[110] Corradi V, Mendez-Villuendas E, Ingólfsson HI, Gu R-X, Siuda I, Melo MN, Moussatova A, DeGagné LJ, Sejdiu BI, Singh G, Wassenaar TA, Delgado Magnero K, Marrink SJ, Tieleman DP (2018). Lipid–protein interactions are unique fingerprints for membrane proteins. ACS Centr Sci.

[111] Stansfeld PJ, Jefferys EE, Sansom MSP (2013). Multiscale simulations reveal conserved patterns of lipid interactions with aquaporins. Structure.

[112] Feng Y, Cronan JE (2009). *Escherichia coli* unsaturated fatty acid synthesis: complex transcription of the fabA gene and in vivo identification of the essential reaction catalyzed by FabB. J Biol Chem.

[113] Martinez-Seara H, Róg T, Pasenkiewicz-Gierula M, Vattulainen I, Karttunen M, Reigada R (2007). Effect of double bond position on lipid bilayer properties: insight through atomistic simulations. J Phys Chem B.

[114] Olofsson G, Sparr E (2013). Ionization constants pK a of cardiolipin. PLoS ONE.

[115] Cousins KR (2005). ChemDraw Ultra 9.0. CambridgeSoft, 100 CambridgePark Drive, Cambridge, MA 02140. www. cambridgesoft. com. See Web site for pricing options. J Am Chem Soc.

[116] Wu EL, Cheng X, Jo S, Rui H, Song KC, Dávila-Contreras EM, Qi Y, Lee J, Monje-Galvan V, Venable RM, Klauda JB, Im W (2014). CHARMM-GUI membrane builder toward realistic biological membrane simulations. J Comput Chem.

[117] Páll S, Abraham MJ, Kutzner C, Hess B, Lindahl E, Markidis S, Laure E (2015). Tackling exascale software challenges in molecular dynamics simulations with GROMACS. Solving software challenges for Exascale.

[118] de Jong DH, Baoukina S, Ingólfsson HI, Marrink SJ (2016). Martini straight: boosting performance using a shorter cutoff and GPUs. Comput Phys Commun.

[119] Bussi G, Donadio D, Parrinello M (2007). Canonical sampling through velocity rescaling. J Chem Phys.

[120] Parrinello M, Rahman A (1981). Polymorphic transitions in single crystals: a new molecular dynamics method. J Appl Phys.

[121] Wassenaar TA, Pluhackova K, Böckmann RA, Marrink SJ, Tieleman DP (2014). Going backward: a flexible geometric approach to reverse transformation from coarse grained to atomistic models. J Chem Theory Comput.

[122] Sandoval-Perez A, Pluhackova K, Böckmann RA (2017). A critical comparison of biomembrane force fields: protein-lipid interactions at the membrane interface. J Chem Theory Comput.

[123] Nosé S (1984). A molecular dynamics method for simulations in the canonical ensemble. Mol Phys.

[124] Hoover WG (1985). Canonical dynamics: equilibrium phase-space distributions. Phys Rev A.

[125] Darden T, York D, Pedersen L (1993). Particle mesh Ewald: an N · log(N) method for Ewald sums in large systems. J Chem Phys.

[126] Sui H, Han B-G, Lee JK, Wallan P, Jap BK (2001). Structural basis of water-specific transport through the AQP1 water channel. Nature.

[127] Hu G, Qi L, Dou X, Wang J (2013). The influences of protonation state of histidine on aromatic/arginine region of aquaporin-1 protein. Mol Simul.

[128] Niemelä PS, Hyvönen MT, Vattulainen I (2006). Influence of chain length and unsaturation on sphingomyelin bilayers. Biophys J.

[129] Harrell Jr FE, Dupont C, Others. Hmisc: Harrell Miscellaneous. 2018. https://CRAN.R-project.org/package=Hmisc. R package version 4.1-1.

[130] Research Systems Inc (1995). IDL user’s guide : interactive data language version 4.

[131] Schrödinger, LLC. The PyMOL Molecular Graphics System, Version 1.3r1; 2010.

[132] Hannesschläger C, Barta T, Siligan C, Horner A (2018). Quantification of water flux in vesicular systems. Sci Rep.

[133] Pohl P, Saparov SM, Borgnia MJ, Agre P (2001). Highly selective water channel activity measured by voltage clamp: analysis of planar lipid bilayers reconstituted with purified AqpZ. Proc Natl Acad Sci U S A.

