# Supplementary material for: Native-like membrane models of E. coli polar lipid extract shed light on the importance of lipid composition complexity
Source: BMC Biol. 2021 Jan 13;19:4. doi: 10.1186/s12915-020-00936-8 (PMC7807449; doi:10.1186/s12915-020-00936-8)
Supplement: Supplementary file 1 — Additional file 1 Tables S1-S6 and Figures S1-S25. Table S1 - Lipid tail names, shortcuts and stereochemistry. Table S2 - Molecular composition of each simulation system. Table S3 - List of performed simulations. Table S4 - Angle parameters for Martini3 tails including cyclopropane bead. Table S5 - Details on water permeability at AA resolution. Table S6 - Details on water permeability at CG resolution. Fig. S1 - Chemical structures of the lipid headgroups in E. coli polar extract membranes. Fig. S2 - Chemical structures of lipid tails included in this study. Fig. S3 - ChemDraw representations of lipid molecules in the Avanti model. Fig. S4 - Angle distributions from AA and CG simulations extracted from the Avanti model of the E. coli polar extract membrane simulations at 310 K. Fig. S5 - The time evolution of the average area per lipid of AA pure lipid bilayers. Fig. S6 - The time evolution of the average area per lipid of CG pure lipid bilayers. Fig. S7 - Area per lipid of each lipid type in pure lipid bilayers at all temperatures and their temperature dependence. Fig. S8 - Statistical analysis of error estimation of APL. Fig. S9 - Ratios of the curved area per lipid and the area per lipid estimated form the box size for all systems at all temperatures. Fig. S10 - Distribution of hydrophobic thickness of 171 transmembrane proteins localized in the inner membrane of E. coli obtained from the OPM database. Fig. S11 - Lipid diffusion in each simulated system for each lipid type including the corresponding activation energies. Fig. S12 - Interdigitation of membrane leaflets. Fig. S13 - Lipid mixing at low (277 K) and high (310 K) temperature visualized by coloring lipids according to their headgroups. Fig. S14 - Lipid tail mixing in individual leaflets at low (277K) and high (310K) temperature visualized by coloring different lipid tail types in different colors. Fig. S15 - Enrichment and depletion of neighboring lipids. Fig. S16 - CL clustering at different temperatu [file 12915_2020_936_MOESM1_ESM.pdf]

Additional file 1: Native-like membrane models of *E. coli* polar lipid extract shed light on the importance of lipid composition complexity

Kristyna Pluhackova*,† and Andreas Horner‡

†*Department of Biosystems Science and Engineering, Eidgenössiche Technische Hochschule (ETH) Zürich, 4058 Basel, Switzerland*

‡*Institute of Biophysics, Johannes Kepler University Linz, Gruberstrasse 40, 4020 Linz, Austria*

E-mail: kristyna.pluhackova@bsse.ethz.ch

Table S1: Lipid tail names, shortcuts and stereochemistry.

P	16:0	palmitoyl
M	cy17:0^{cis9,10}	cis-9,10-methylene-hexadecanoic-acid
N	cy19:0^{cis11,12}	cis-11,12-methylene-octadecanoic-acid
V	18:1^{cis11,12}	cis-11,12-octadecenoic-acid
Y	16:1^{cis9,10}	palmitoleic acid
O	18:1^{cis9,10}	oleic acid
J	cy17:0^{cis11,12}	cis-11,12-methylene-hexadecanoic-acid

Lipid tails that are not native to *E. coli* PLE but were used in the simple models for comparative purposes are colored grey.

Table S2: Molecular composition of each simulation system.

System	Lipids in each leaflet	Water	Na	AQP1
Avanti	2 YPMN-CL, 2 YPMV-CL, 4 MPPV-CL, 1 MPPM-CL<br>24 PMPG, 4 PNPG, 2 YVPG, 8 PVPG, 4 PYPG<br>28 YVPE, 34 PMPE, 11 PNPE, 17 MVPE, 52 PVPE	16364	120	
Avanti CG	2 YPMN-CL, 2 YPMV-CL, 4 MPPV-CL, 1 MPPM-CL<br>24 PMPG, 4 PNPG, 2 YVPG, 8 PVPG, 4 PYPG<br>28 YVPE, 34 PMPE, 11 PNPE, 17 MVPE, 52 PVPE	4091	120	
SimplePOM	9 MPPO-CL, 42 MMPG, 142 POPE	17924	120	
SimplePOM CG	9 MPPO-CL, 42 MMPG, 142 POPE	4481	120	
SimplePVM	9 MPPV-CL, 42 MMPG, 142 PVPE	18404	120	
SimplePVM CG	9 MPPV-CL, 42 MMPG, 142 PVPE	4601	120	
SimplePVJ	9 JPPV-CL, 42 JJPG, 142 PVPE	18404	120	
SimplePVJ CG	9 JPPV-CL, 42 JJPG, 142 PVPE	4601	120	
AQP1 in *Avanti*	2 YPMN-CL, 2 YPMV-CL, 4 MPPV-CL, 1 MPPM-CL<br>24 PMPG, 4 PNPG, 2 YVPG, 8 PVPG, 4 PYPG<br>28 YVPE, 34 PMPE, 11 PNPE, 17 MVPE, 52 PVPE	28991	104	4
AQP1 CG in *Avanti* CG	2 YPMN-CL, 2 YPMV-CL, 4 MPPV-CL, 1 MPPM-CL<br>24 PMPG, 4 PNPG, 2 YVPG, 8 PVPG, 4 PYPG<br>28 YVPE, 34 PMPE, 11 PNPE, 17 MVPE, 52 PVPE	7379	104	4

Note, that the Martini model of O and Y lipid tail is the same, as well as N and J models equal and that the coarse-grained water model used here represents 4 atomistic water molecules each. AQP1 stands for an AQP1 monomer.

Table S3: List of performed simulations

System	Resolution	Temperature [K]	Simulation length [μs]
Avanti<br>*SimplePOM*	Martini3	277	1×10
		285	1×10
		296	1×10
		310	1×10
		320	1×10
SimplePVM<br>*SimplePVJ*	Martini3	277	1×5
		285	1×5
		296	1×5
		310	1×5
		320	1×5
Avanti	CHARMM36	277	2×1
		285	4×0.5
		296	2×0.5
		310	2×0.5
		320	2×0.5
SimplePOM	CHARMM36	277	2×1
		285	3×0.5
		296	2×0.5
		310	3×0.5
		320	2×0.5
SimplePVM<br>*SimplePVJ*	CHARMM36	277	1×0.5
		285	1×0.5
		296	1×0.5
		310	1×0.5
		320	1×0.5
AQP1 in *Avanti*	Martini3	277	1×10
		285	1×10
		296	1×10
		310	1×10
AQP1 in *Avanti*	CHARMM36	277	1×0.5
		289	1×0.5
		296	1×0.5
		309	1×0.5

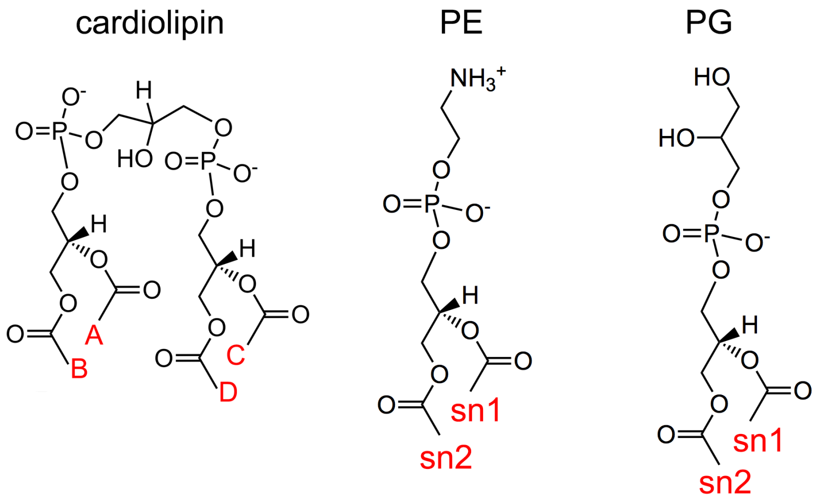

Figure S1: Chemical structures of the lipid headgroups in *E. coli* polar extract membranes. Lipid tail positions are highlighted in red, with shortcuts following schemes ABCD-CL, sn2sn1PE, and sn2sn1PG. Pictures were generated using ChemDraw JS.

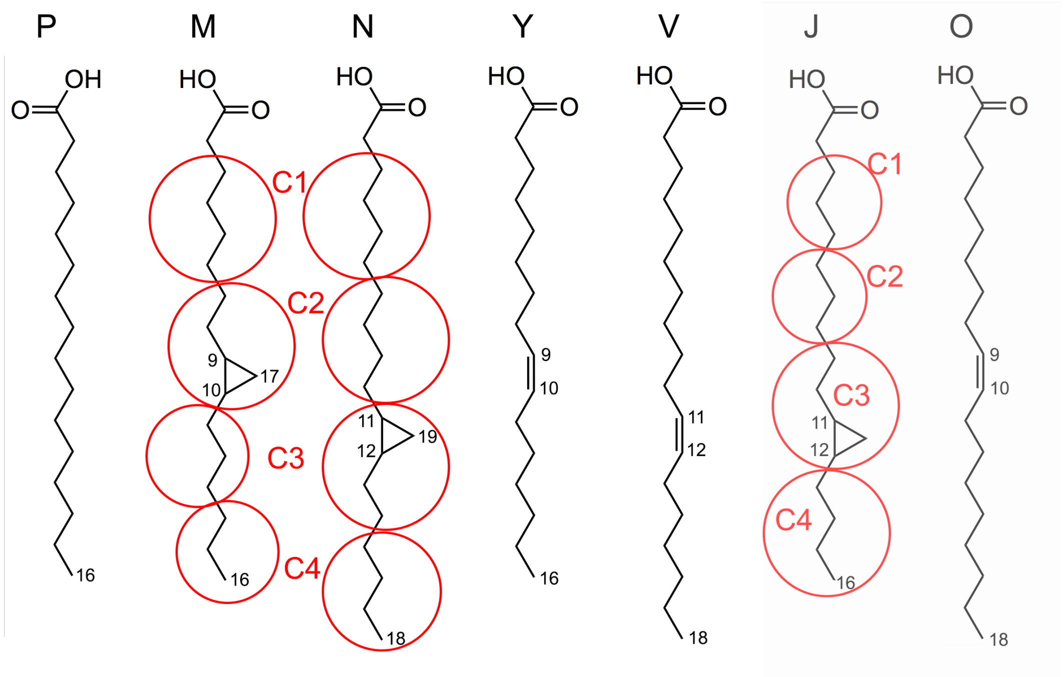

Figure S2: Chemical structures of lipid tails included in this study. For tails including cyclopropane units the Martini3 mapping is shown in red. The lipid tails J and O which were used in *Simple* models for test purposes only are partially transparent. Please note that in the Martini3 force field Y and O tails are represented by equal parameters, and N and J are represented by the same parameters. Pictures were generated using ChemDraw JS.

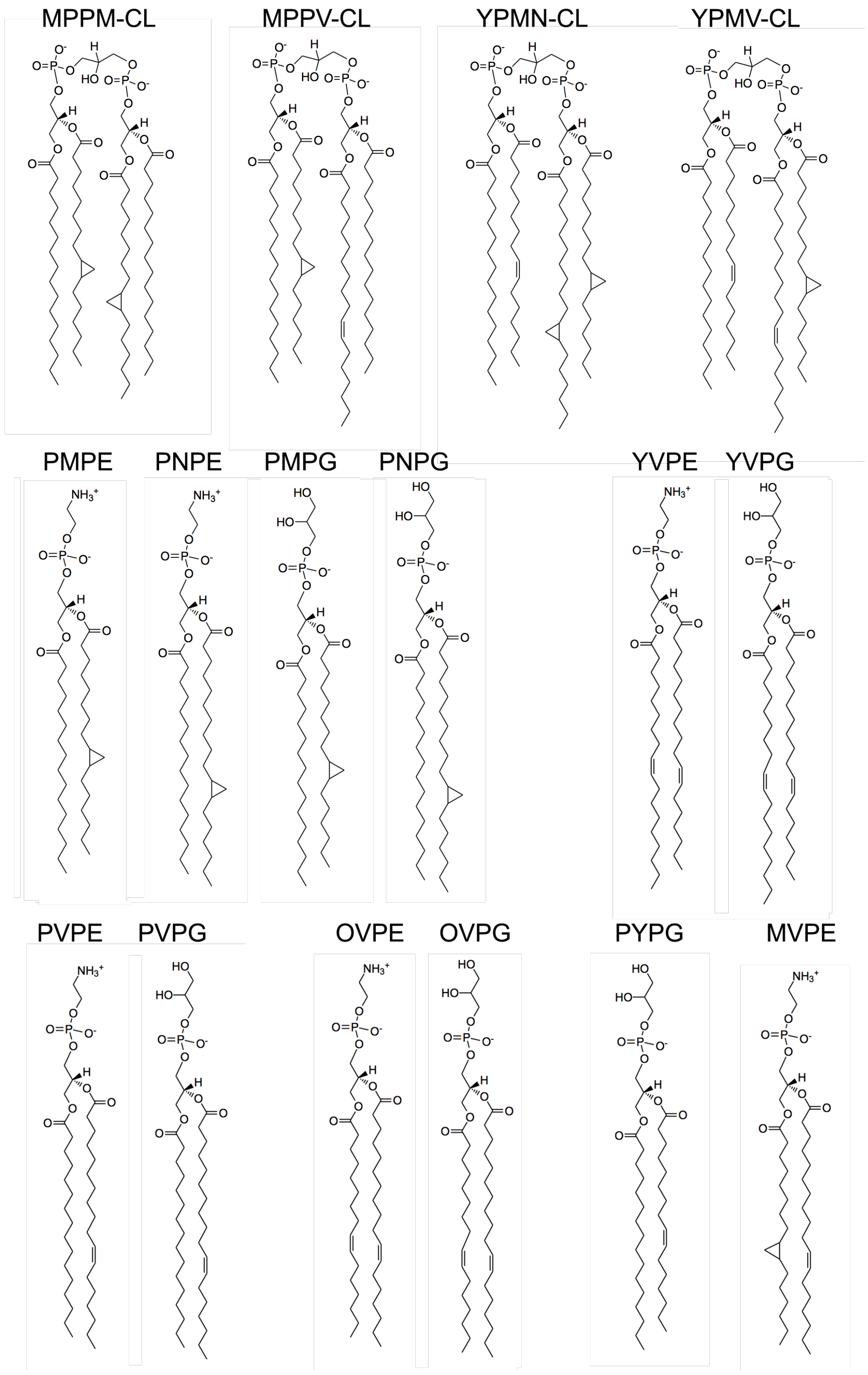

Figure S3: ChemDraw representations of lipid molecules in the *Avanti* model.

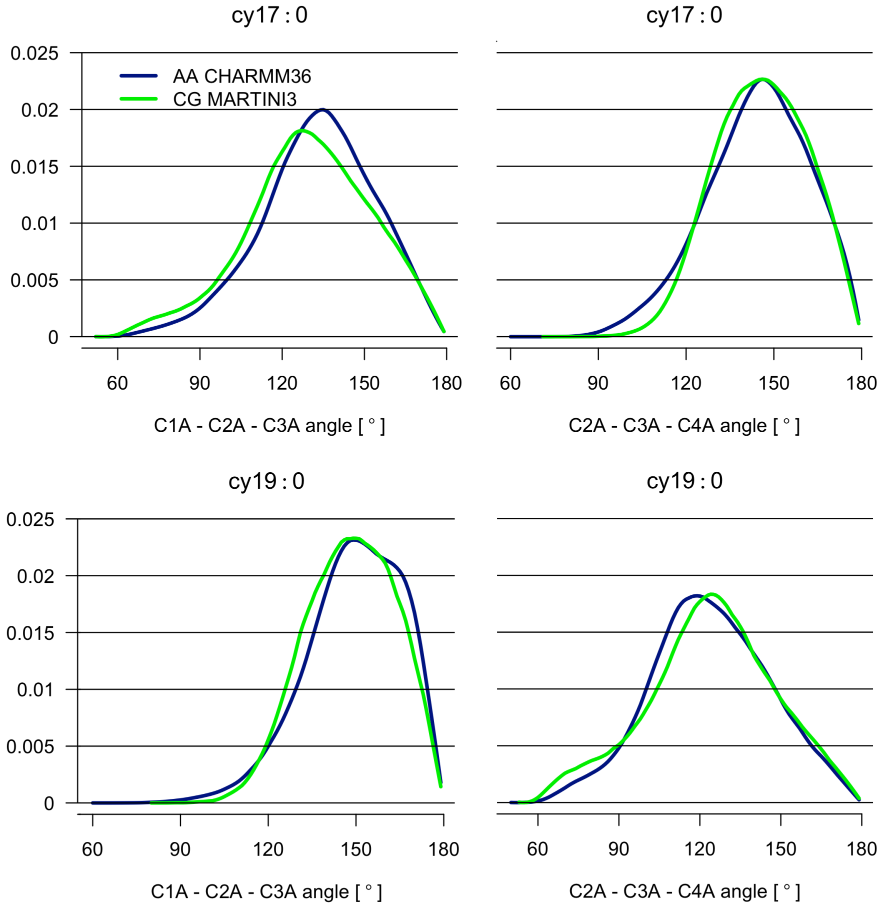

Figure S4: Angle distributions from AA (in dark blue) and CG (in green) simulations extracted from the *Avanti* model of the *E. coli* polar extract membrane simulations at 310 K.

Table S4: Angle parameters for Martini3 tails including cyclopropane bead (type C1, highlighted in bold).

Lipid tail	Angle	Equilibrium value [°]	Force constant [kJ/mol/nm^2]
cy17:0	GL1-C1A-**C2A**[b]	180.0	35.0
	C1A-**C2A**-C3A	125.0	15.0
	**C2A**-C3A-C4A[b]	180.0	35.0
cy19:0	GL1-C1A-C2A[a]	180.0	35.0
	C1A-C2A-**C3A**[b]	180.0	35.0
	C2A-**C3A**-C4A	125.0	15.0

[a] Original Martini3 parameter not investigated here. [b] Parameter that appeared to agree perfectly with Martini3 parameter.

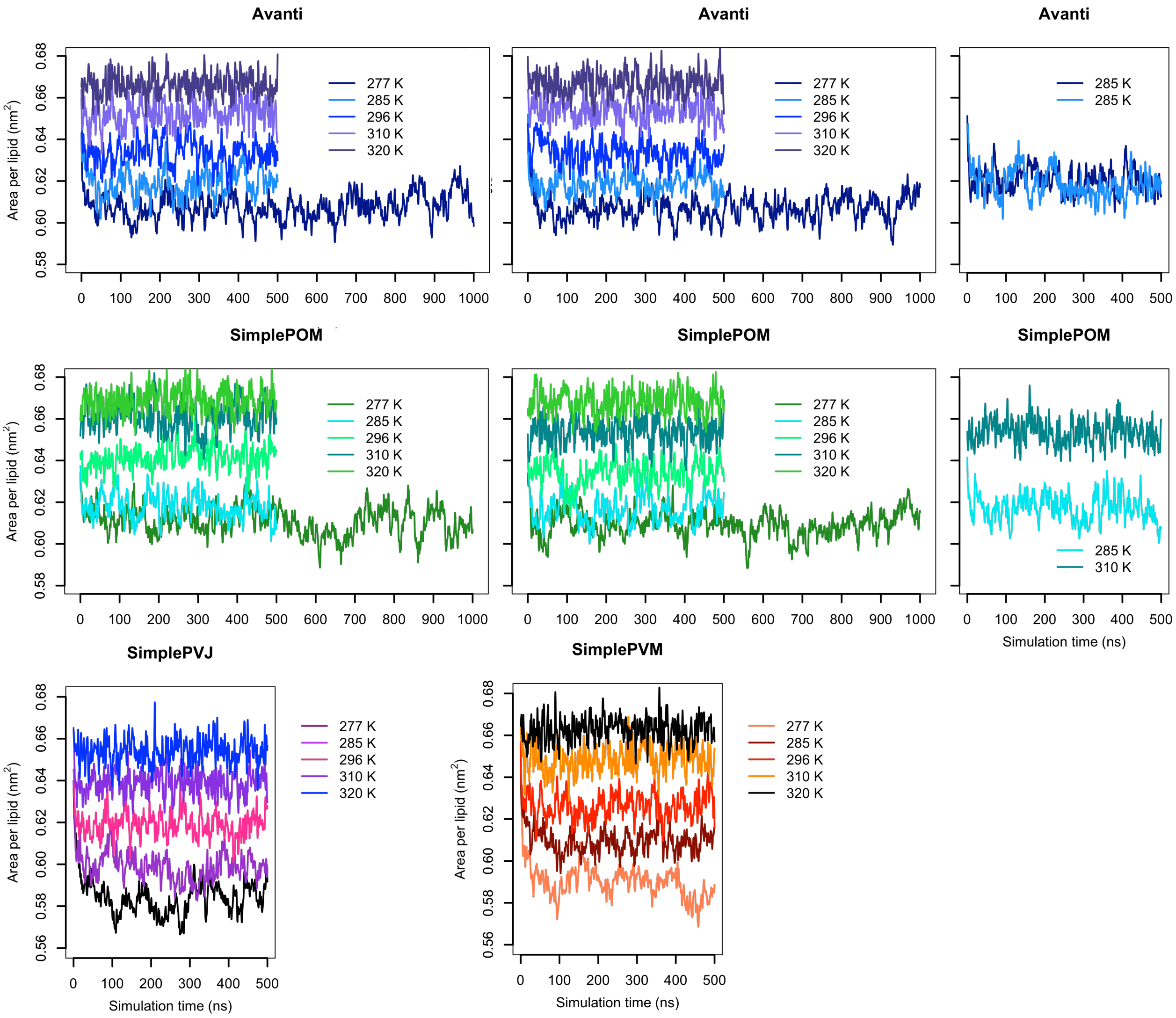

Figure S5: The time evolution of the average area per lipid of AA pure lipid bilayers. AA systems show larger fluctuations of the APL than CG systems (shown in Figure S6). Most simulations equilibrated well within the initial 100 ns of the production run simulations, a timespan which was omitted from analysis (please note that for simulations performed at 277 K even longer time interval, i.e. the initial 200 ns, were excluded from the analysis). Please note the large APL fluctuations of the *SimplePVM* system at 277 K and *SimplePVJ* system at 277 K and 285 K.

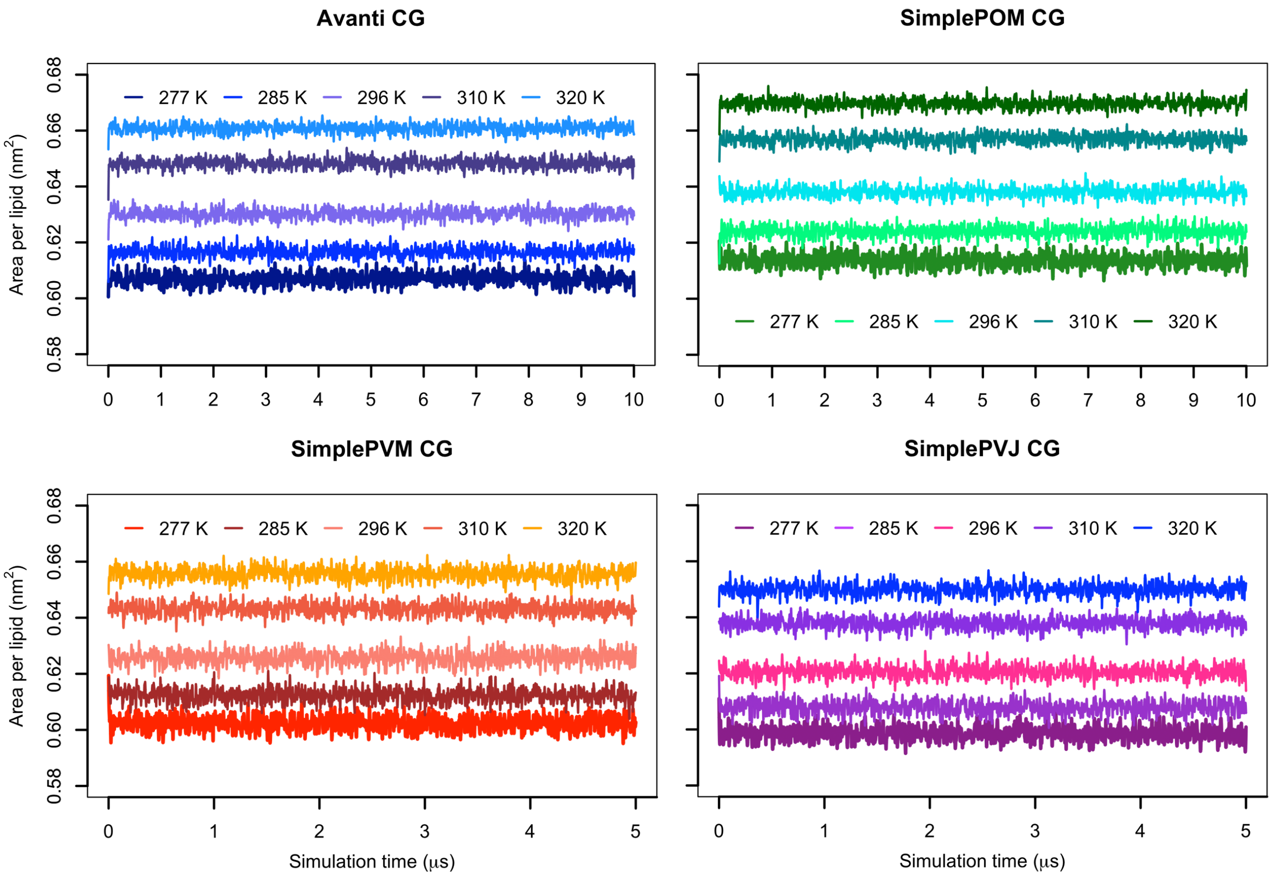

Figure S6: The time evolution of the average area per lipid of CG pure lipid bilayers.

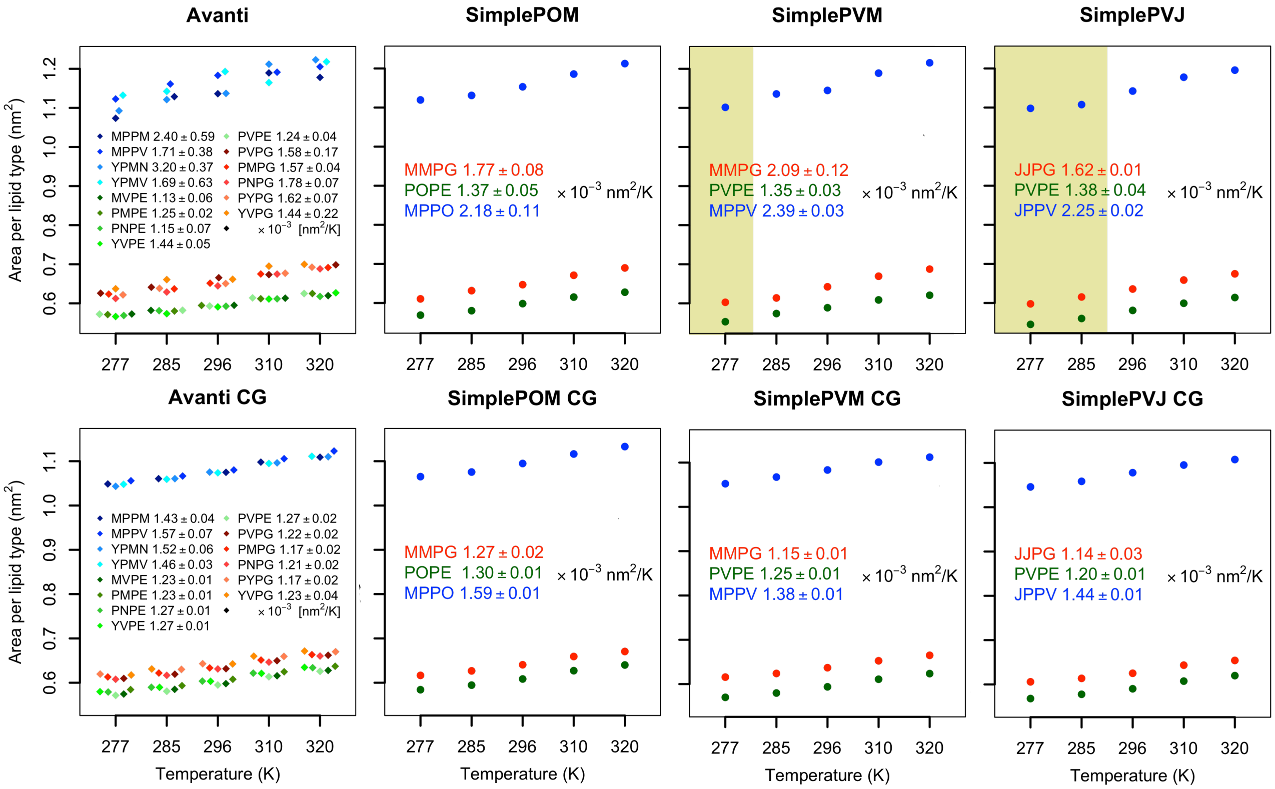

Figure S7: Area per lipid of each lipid type, calculated by means of the Voronoi tenselation by the tool APL@Voro, in pure lipid bilayers at all temperatures and their temperature dependence. The values not included in the fit, due to the partial transition into the liquid-ordered state, have yellow background.

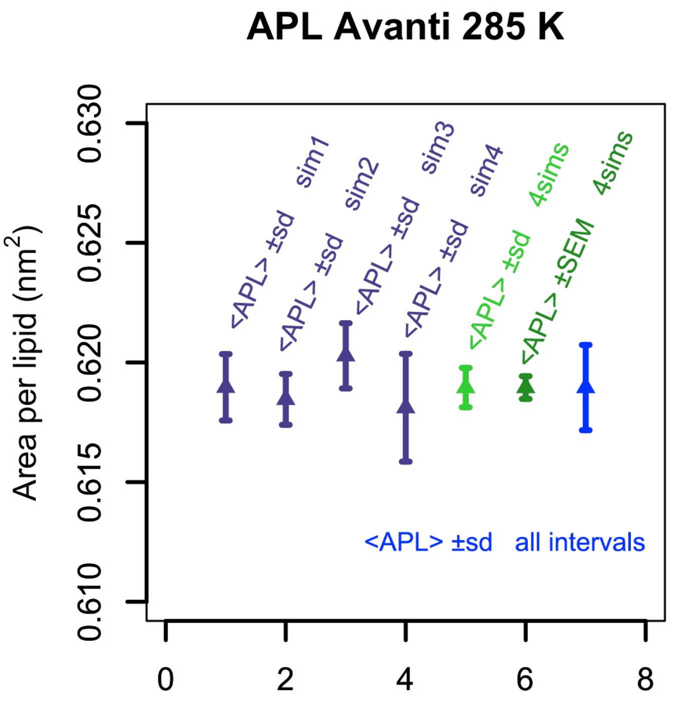

Figure S8: Statistical analysis of error estimation of APL, a typical membrane structural property. From each 500 ns long simulation, the last 400 ns were analyzed, either as whole or split into 100 ns intervals. sd stands for standard deviation, SEM for standard error of the mean. "4sims" denote an average over whole simulations and "all intervals" an average over all, i.e. sixteen, 100 ns long intervals. This comparison serves to evaluate the effect of using block averages for error estimation of typical membrane structural properties statistical analysis of replicate simulations. For this test, the area per lipid of an *Avanti* membrane model at 285 K was chosen because APL is a typical membrane property which fluctuates over the time and at lower temperatures can take longer to achieve an equilibrated value. The results have shown, that block averaging rather overestimates the error, as compared to averages over whole simulations. However, the differences between the average values, if including the error bars, are insignificant.

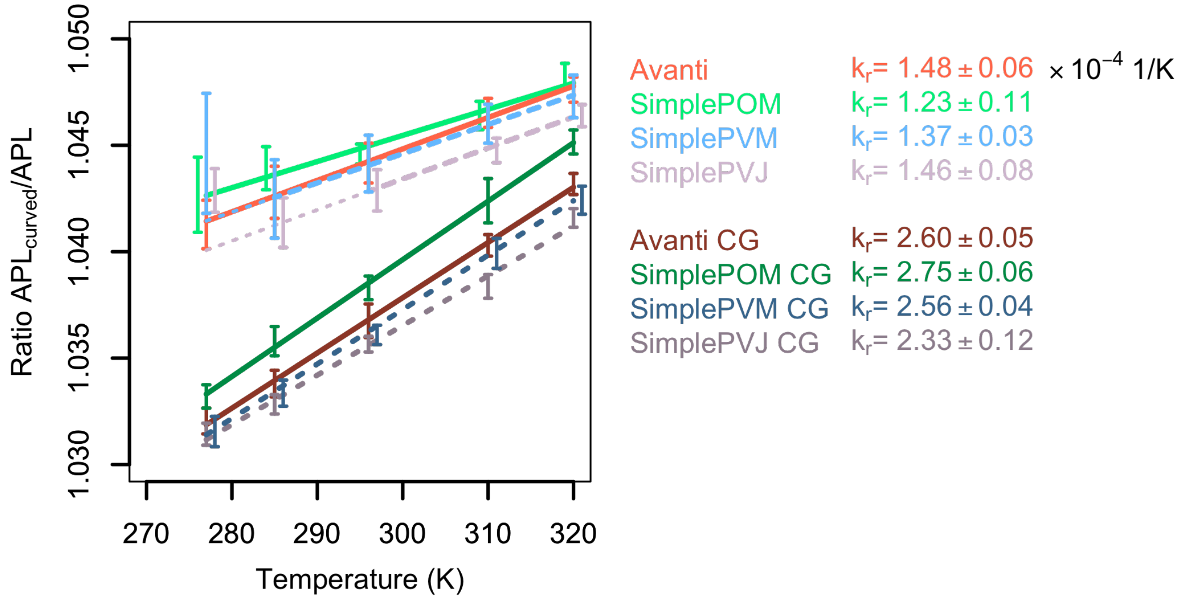

Figure S9: Ratios of the curved area per lipid and the area per lipid estimated form the box size for all systems at all temperatures. The atomistic models show higher ratio of curved and standard area per lipid (APL) hinting to larger membrane curvature. On the other hand, the temperature dependence of the ratio is significantly weaker in the atomistic models. The *SimplePOM* lipids in each resolution (i.e. AA and CG) give the largest APL ratio, while the *SimplePVJ* model is the least curved of all models at the given resolution. The APL ratio of the *SimplePVM* and the *Avanti* models are comparable. The increased ratios of the *SimplePVM* and *SimplePVJ* models at 277 K at AA resolution is another indicator of a partial phase transition of the system to the liquid ordered phase.

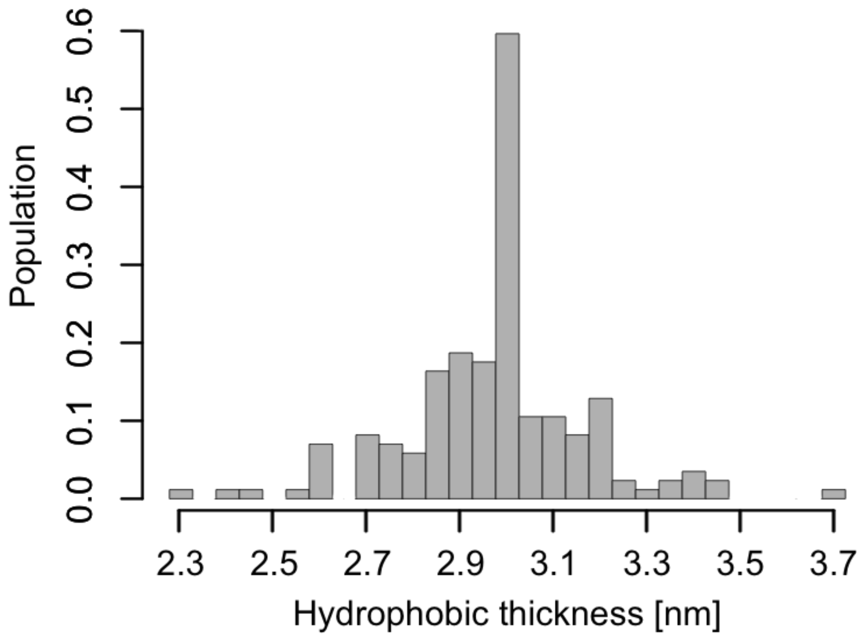

Figure S10: Distribution of hydrophobic thicknesses of 171 transmembrane proteins localized in the inner membrane of *E. coli*. The thicknesses are extracted from the OPM database. The average hydrophobic thickness amounts to 2.97±0.19 nm.

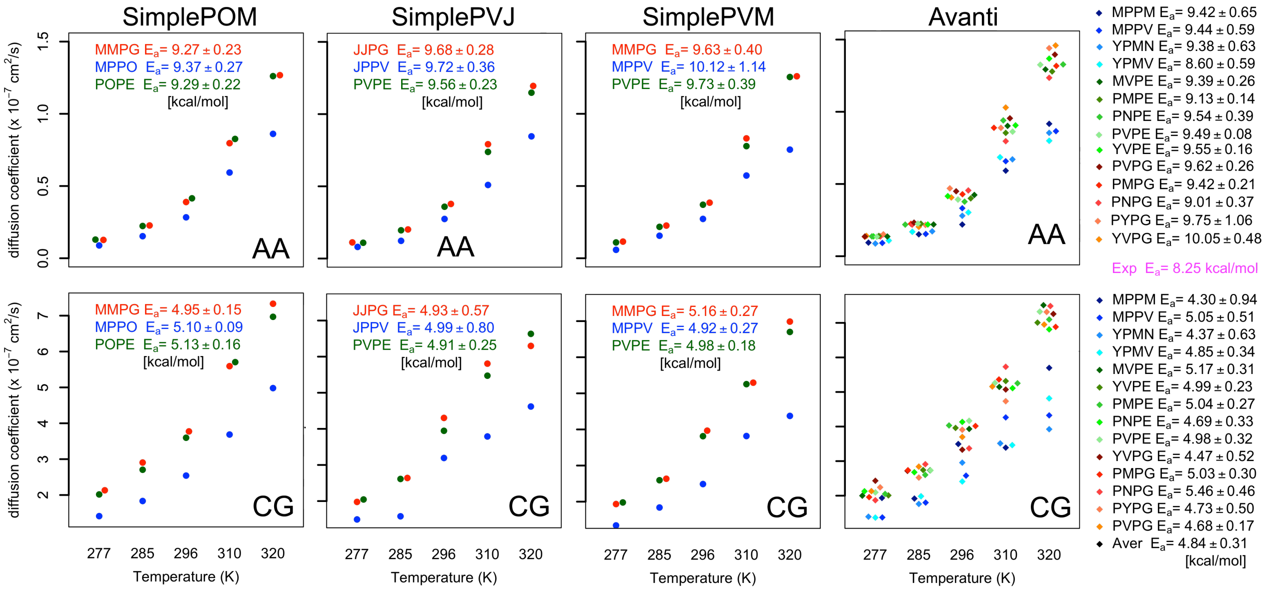

Figure S11: Lipid diffusion in each simulated system for each lipid type including the corresponding activation energies. The self-diffusion coefficients of each lipid type are color coded and plotted as a single dot each. The R's Beeswarm function was used to create one dimensional scatter plots for each temperature.

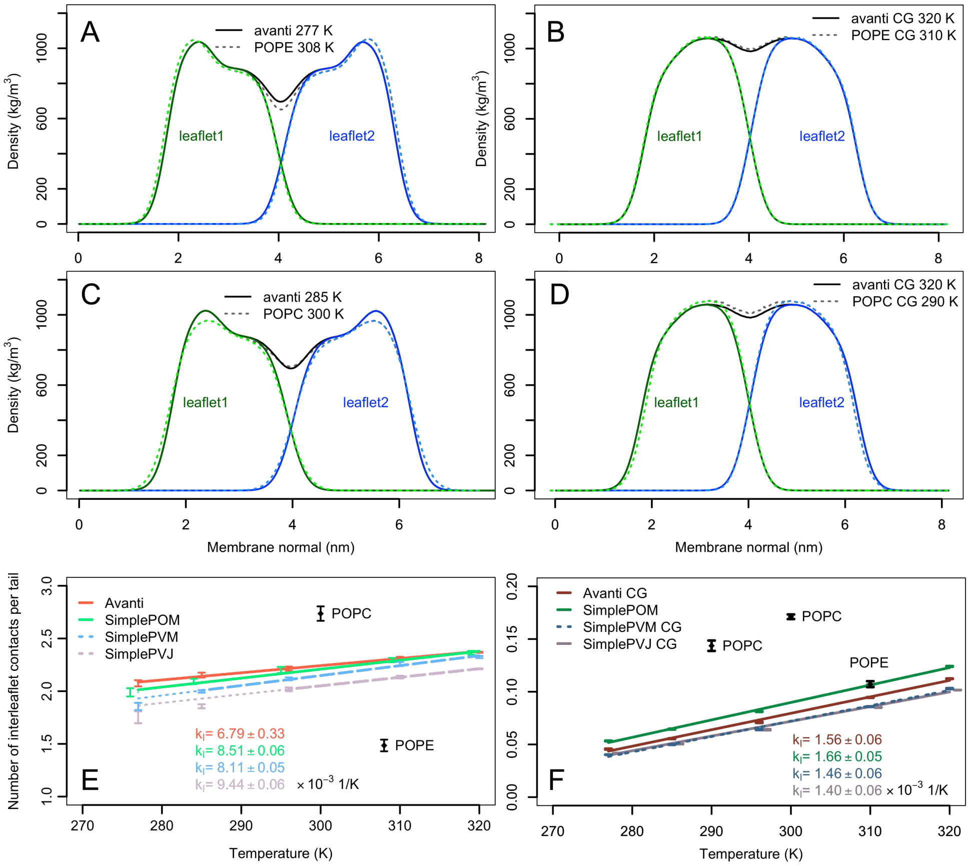

Figure S12: Interdigitation visualised as overlapping leaflet densities (A-D) and quantified as contacts among the lipid tails between the two leaflets excluding the terminal methyl group or C4 beads (E and F, respectively). Interdigitation properties of pure POPC and POPE bilayers of the same size and simulated using the same force field were included for comparison. For density profiles, membranes of similar thickness were chosen for comparison instead of the same temperature.

Table S5: Details on water permeability at AA resolution.

System	Temperature [K]	Analyzed time [μs]	Passed waters per sim	Water volume [nm^3]	Flux [μm/s]
	277	2x0.9	5.5±2.12	0.02923	1.52±0.59
	285	4x0.4	8.25±2.18	0.02923	5.07±1.34
Avanti	296	2x0.4	19±2.83	0.02941	11.53±1.73
	310	2x0.4	54.5±3.53	0.02967	32.46±2.07
	320	2x0.4	138.5±3.53	0.03004	81.63±2.13
	277	2x0.9	7.5±3.53	0.02923	2.09±0.99
	285	2x0.4	5.3±2.27	0.02923	3.29±1.41
SimplePOM	296	2x0.4	14±2.83	0.02941	8.47±1.69
	310	2x0.4	65±1.87	0.02967	38.68±1.10
	320	2x0.4	125±16.97	0.03004	73.45±9.88
	277	0.4	1	0.02923	0.64
	285	0.4	9	0.02923	5.01
SimplePVM	296	0.4	23	0.02941	14.11
	310	0.4	59	0.02967	35.37
	320	0.4	130	0.03004	77.02
	277	0.4	1	0.02923	0.65
	285	0.4	3	0.02923	1.91
SimplePVJ	296	0.4	16	0.02941	9.93
	310	0.4	48	0.02967	29.22
	320	0.4	124	0.03004	74.44

Water volume corresponds to the volume of one water molecule. The errors denote standard errors of the mean obtained from averages over individual simulations. The first 100 ns were excluded from the analysis for equilibration purposes. The gray colored values were excluded from the Arrhenius fit, due to too low credibility of the data (less than one water passage per 100 ns).

Table S6: Details on water permeability at CG resolution.

System	Temperature [K]	Analyzed time [μs]	Passed waters per μs	Water volume [nm^3]	Flux [μm/s]
Avanti CG	277	9x4*	13.33±3.46	0.11831	3.37±0.87
	285	9x4*	21.00±2.79	0.11926	5.26±0.70
	296	9x4*	47.33±1.07	0.12056	11.73±0.27
	310	9x4*	114.11±7.06	0.12224	27.87±1.72
	320	9x4*	195.50±12.53	0.12353	47.34±3.03
SimplePOM CG	277	9x4*	12.33±2.36	0.11831	3.08±0.59
	285	9x4*	20.44±3.74	0.11926	5.06±0.93
	296	9x4*	44.56±5.54	0.12056	10.90±1.36
	310	9x4*	114.67±6.77	0.12224	27.64±1.63
	320	9x4*	194.86±6.42	0.12353	46.56±1.53
SimplePVM CG	277	4x4*	13.00±4.74	0.11831	3.31±1.21
	285	4x4*	20.00±2.92	0.11926	5.05±0.74
	296	4x4*	40.50±8.14	0.12056	10.11±2.03
	310	4x4*	120.75±4.66	0.12224	29.73±1.15
	320	4x4*	202.00±5.48	0.12353	49.30±1.34
SimplePVJ CG	277	4x4*	11.75±1.09	0.11831	3.01±0.28
	285	4x4*	17.25±4.97	0.11926	4.38±1.26
	296	4x4*	43.25±3.77	0.12056	10.88±0.95
	310	4x4*	106.00±10.89	0.12224	26.31±2.70
	320	4x4*	183.25±12.03	0.12353	45.12±2.96

*The CG simulation time was multiplied by a factor of 4 corresponding to the speed up of CG simulations due to smaller friction of beads as compared to atoms.

Water volume corresponds to the volume of one water bead. The errors denote standard deviations over 1 μs time intervals of CG simulations. The first 1 μs was excluded from the analysis for equilibration purposes.

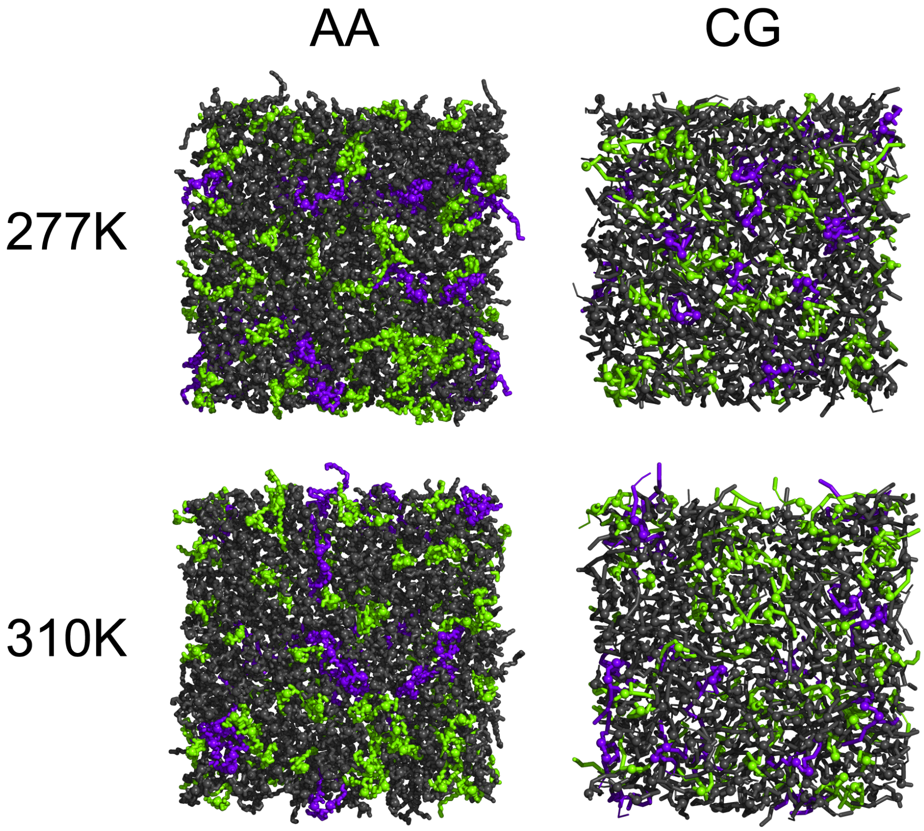

Figure S13: Lipid mixing at low (277 K) and high (310 K) temperature visualized by coloring lipids according to their headgroups. Cardiolipins (CLs) are colored purpleblue, phosphatidylethanolamines (PEs) in dark grey and phosphatidylglycerols (PGs) in green. The atomistic systems at 1000 ns (277 K) and at 500 ns (310 K) are shown on the left and the CG systems after 10μs at equal temperatures are shown on the right. The phosphorus atom or the phosphate bead are shown as spheres.

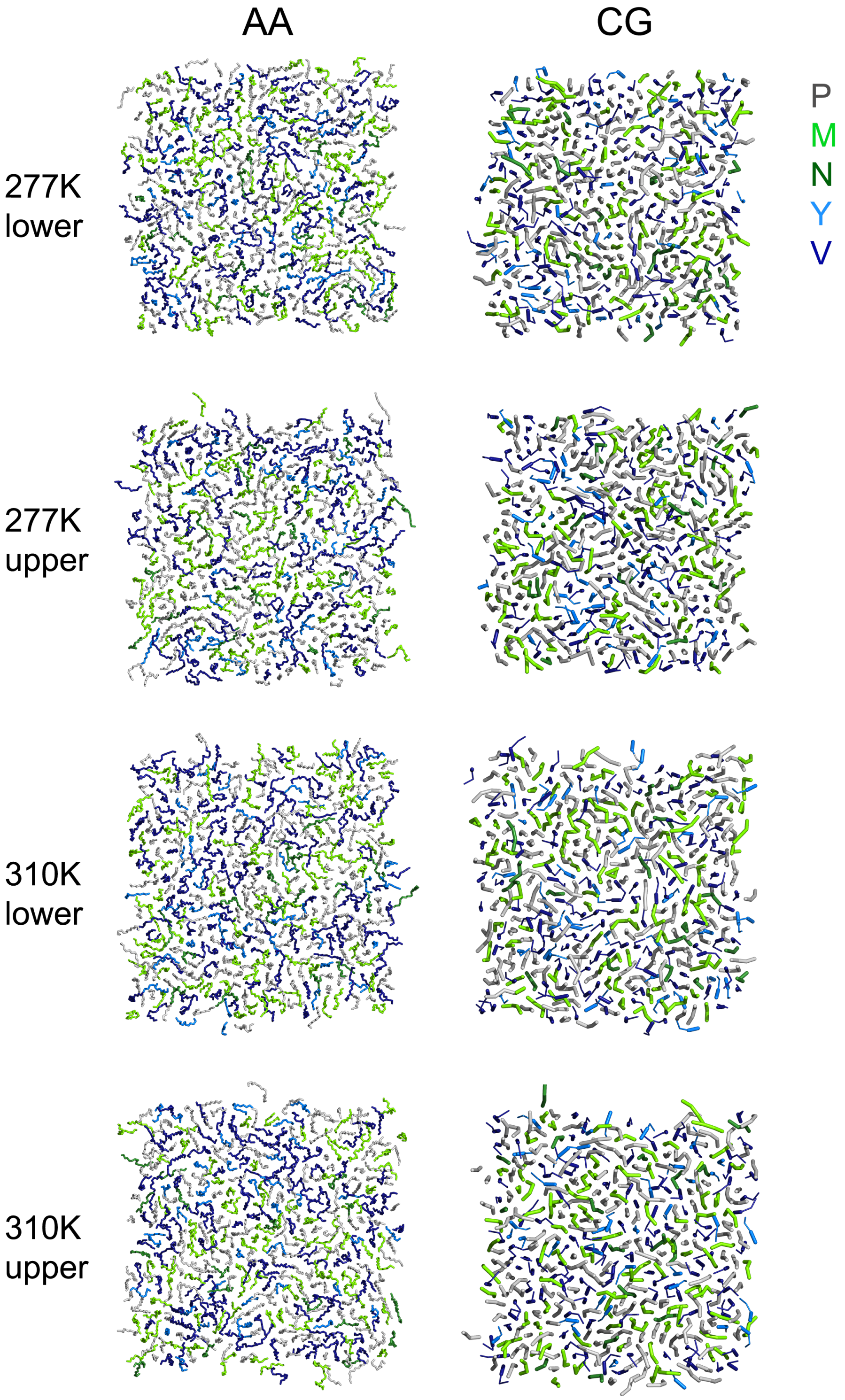

Figure S14: Lipid tail mixing in individual leaflets at low (277 K) and high (310 K) temperature visualized by coloring different lipid tail types in different colors. Palmitoyl tail (P) is colored grey, cy17:0 (M) green, cy19:0 (N) dark green, 16:1 (Y) marine, and 18:1 (V) dark blue.

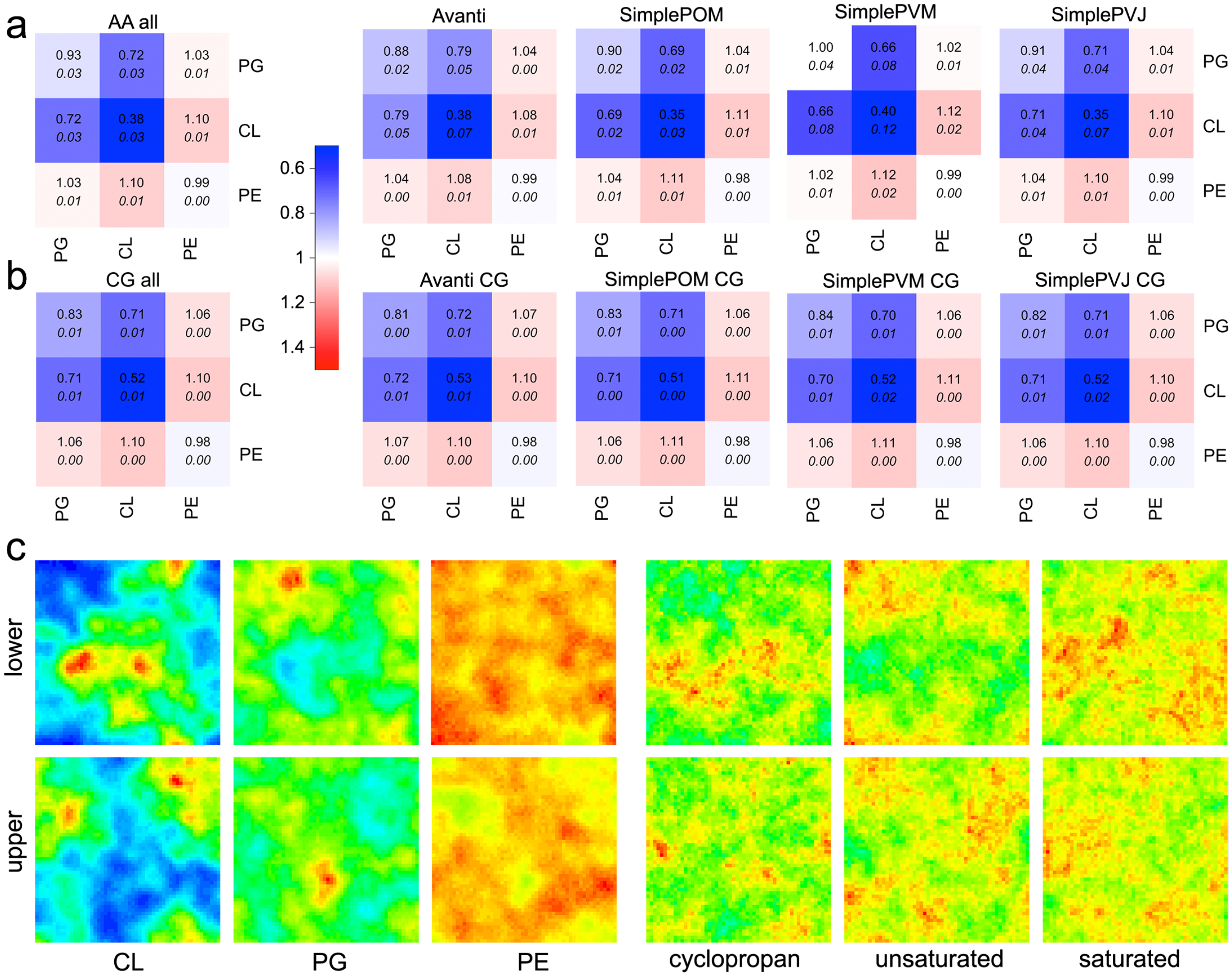

Figure S15: Enrichment and depletion of neighboring lipids. a. Relative enrichment and depletion evaluated as the number of neighboring lipids weighted by the relative composition of those lipids averaged over all AA systems and temperatures (left) and for individual AA systems averaged over all temperatures. b. Relative enrichment and depletion evaluated as the number of neighboring lipids weighted by the relative composition of those lipids averaged over all CG systems and temperatures (left) and for individual CG systems averaged over all temperatures. c. 2D density maps of headgroups (left) and lipid tails (right) in individual leaflets over 9-10 μs of the *Avanti* CG simulation at 277 K. Rainbow scale from blue (0) to red (maximum) density.

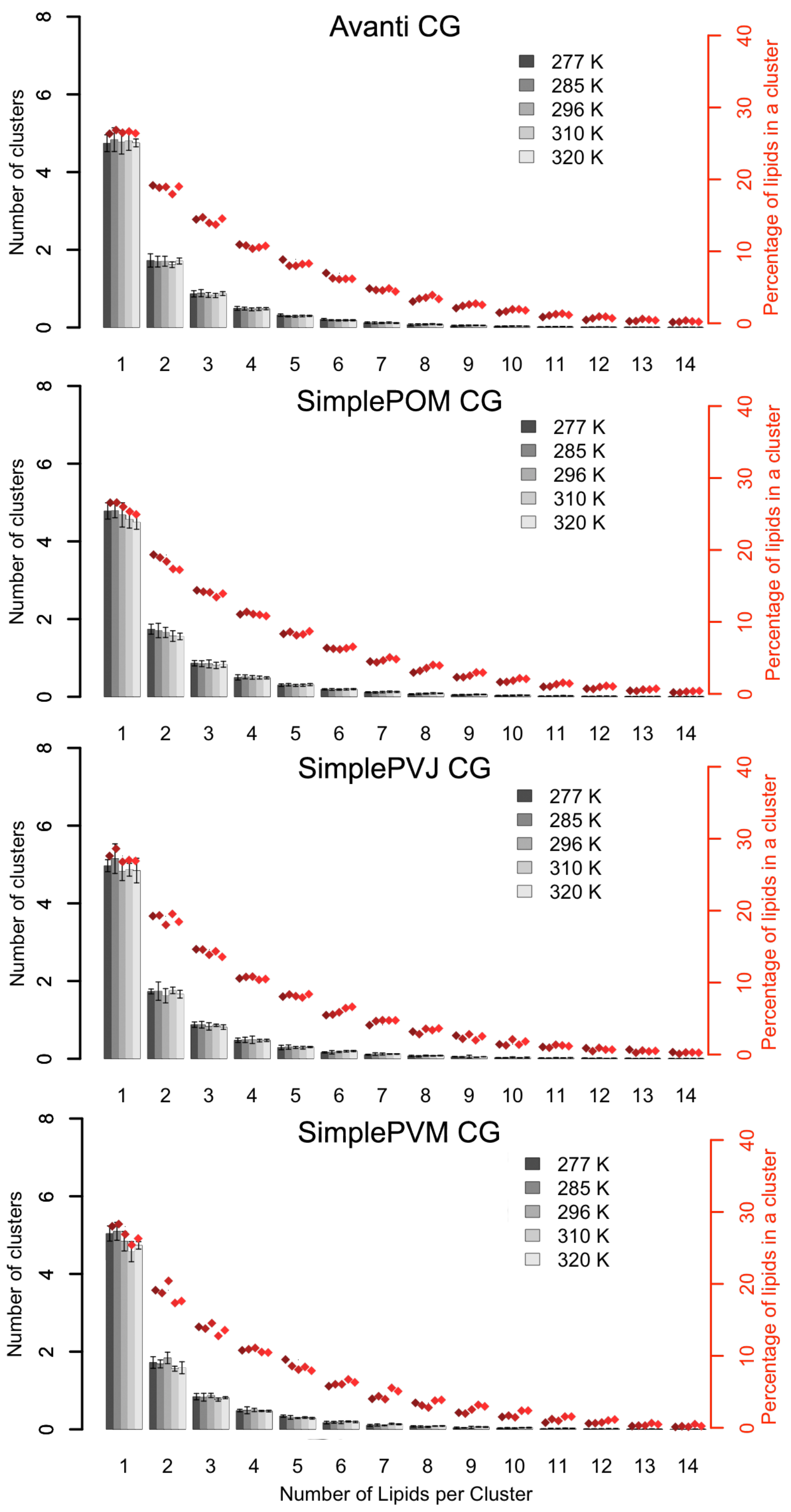

Figure S16: CL clustering at different temperatures in all membrane models at CG resolution. Largest distance considered to be in a cluster amounted to 0.65 nm. The error bars denote standard deviations over 1μs intervals of the simulations.

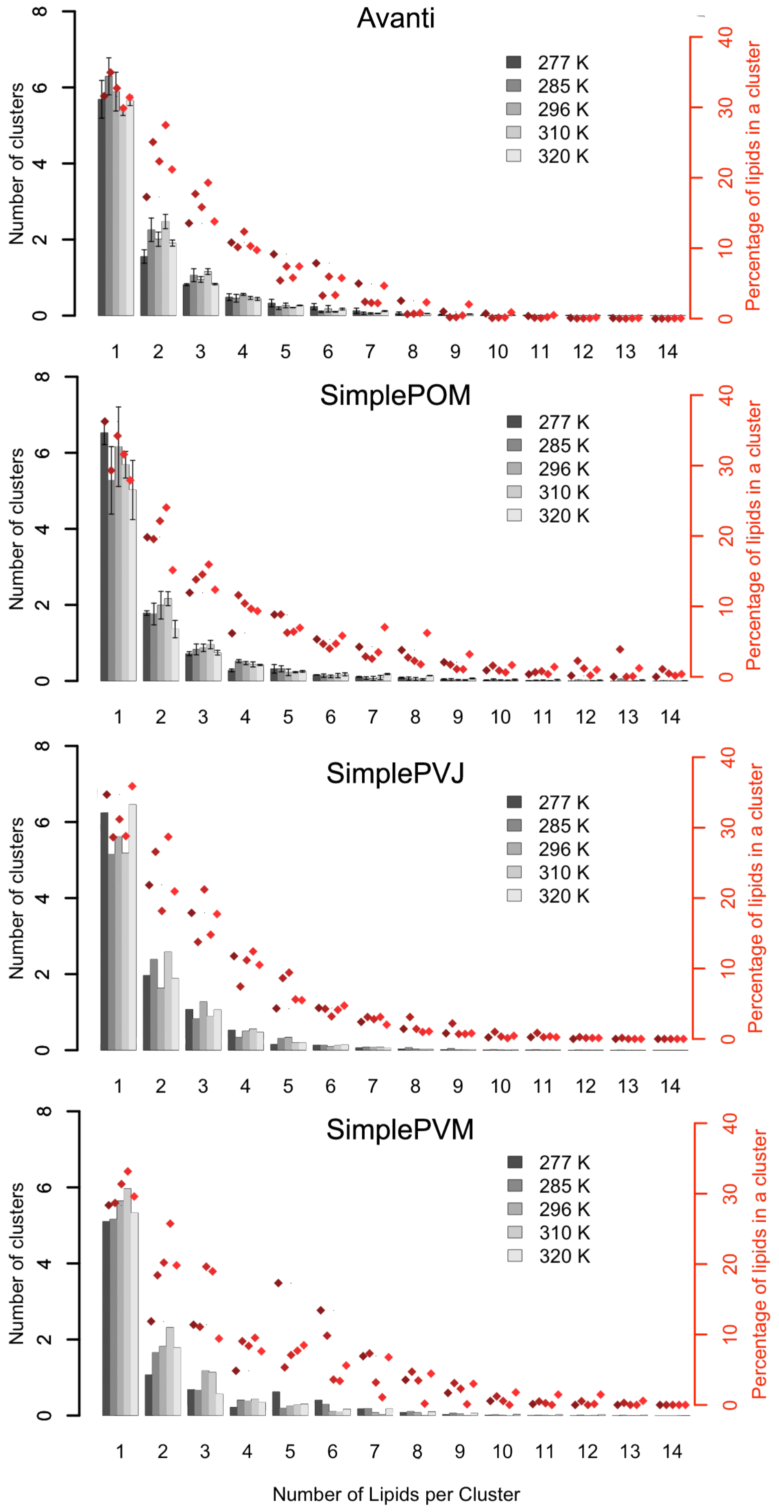

Figure S17: CL clustering at AA resolution at different temperatures for each lipid mixture. Largest distance considered to be in a cluster amounted to 0.35 nm. The error bars give the standard error of the mean over individual simulations.

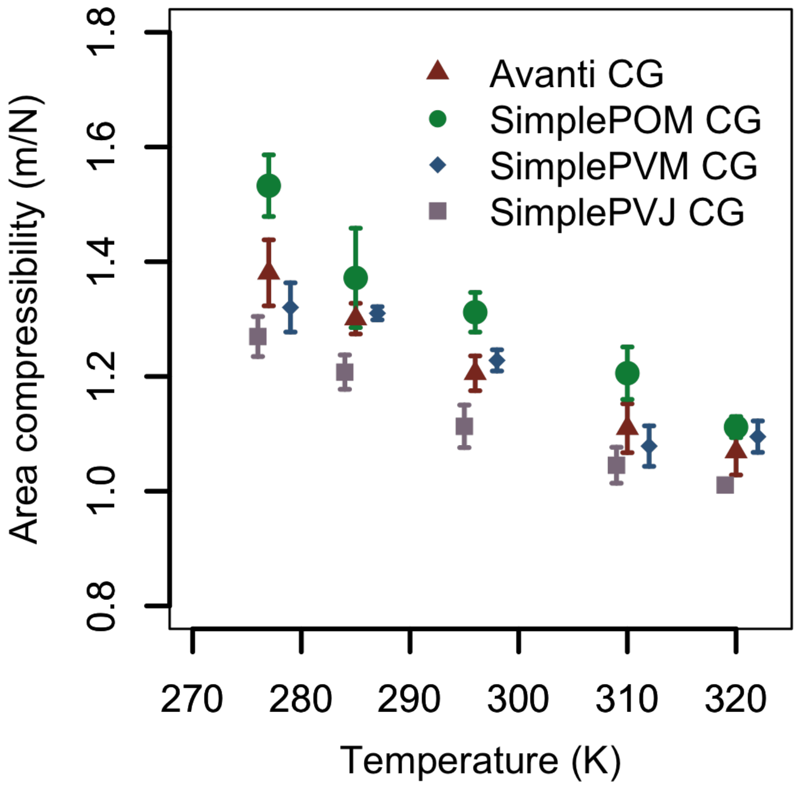

Figure S18: Membrane area compressibility of CG membranes. The compressibility values of the *SimplePVJ* model were moved by 1 K to lower temperature values and of the *SimplePVM* model by 1 K to higher temperature values in order to improve the visibility of the data. The compressibility follows a single albeit nonlinear trend for all membrane models, namely it reduces with increasing temperature, an effect resulting from smaller fluctuations of the average area per lipid at higher temperatures (see Figure S6).

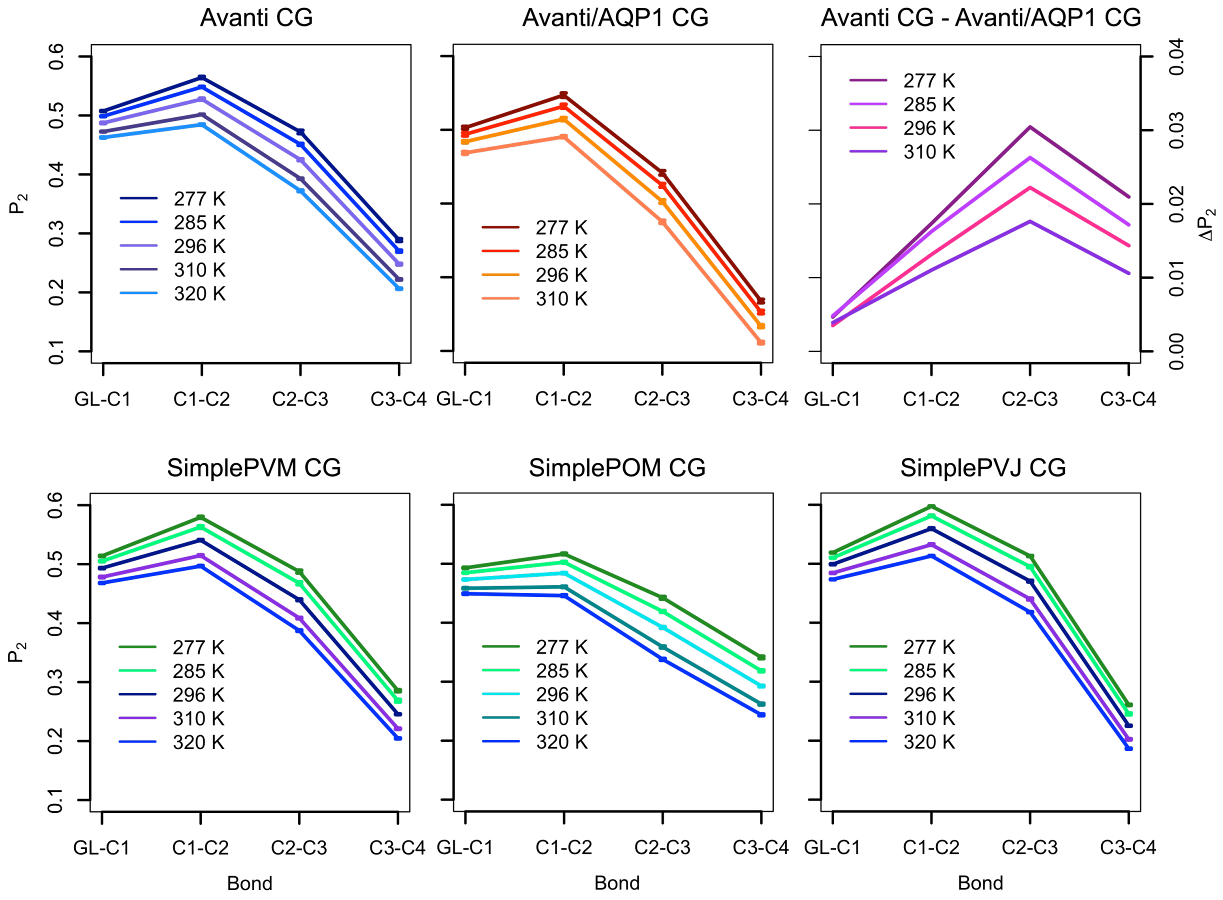

Figure S19: Order parameters along the lipid tails in CG simulations at different temperatures for all four membrane models and for the *Avanti* model including AQP1. "*Avanti* CG - *Avanti*/AQP1 CG" stands for the difference between the *Avanti* systems with and without AQP1. The bonds close to the lipid headgroups are most disordered in the *SimplePOM* CG model, because it contains unsaturations and cyclopropylation at the C2 beads. The inclusion of unsaturation and cyclopropanylation at the C3 beads leads to higher ordering close to the lipid headgroups and to disordering of the last bond in the *Avanti* and *SimplePVM* models. The latter effect is even more pronounced in the *SimplePVJ* CG model. The presence of AQP1 in the *Avanti* CG model reduces the lipid tail order with the exception of the very first bond GL1-C1 which connects the lipid tails to the glycerol backbone, where the AQP1 ordering effect is negligible.

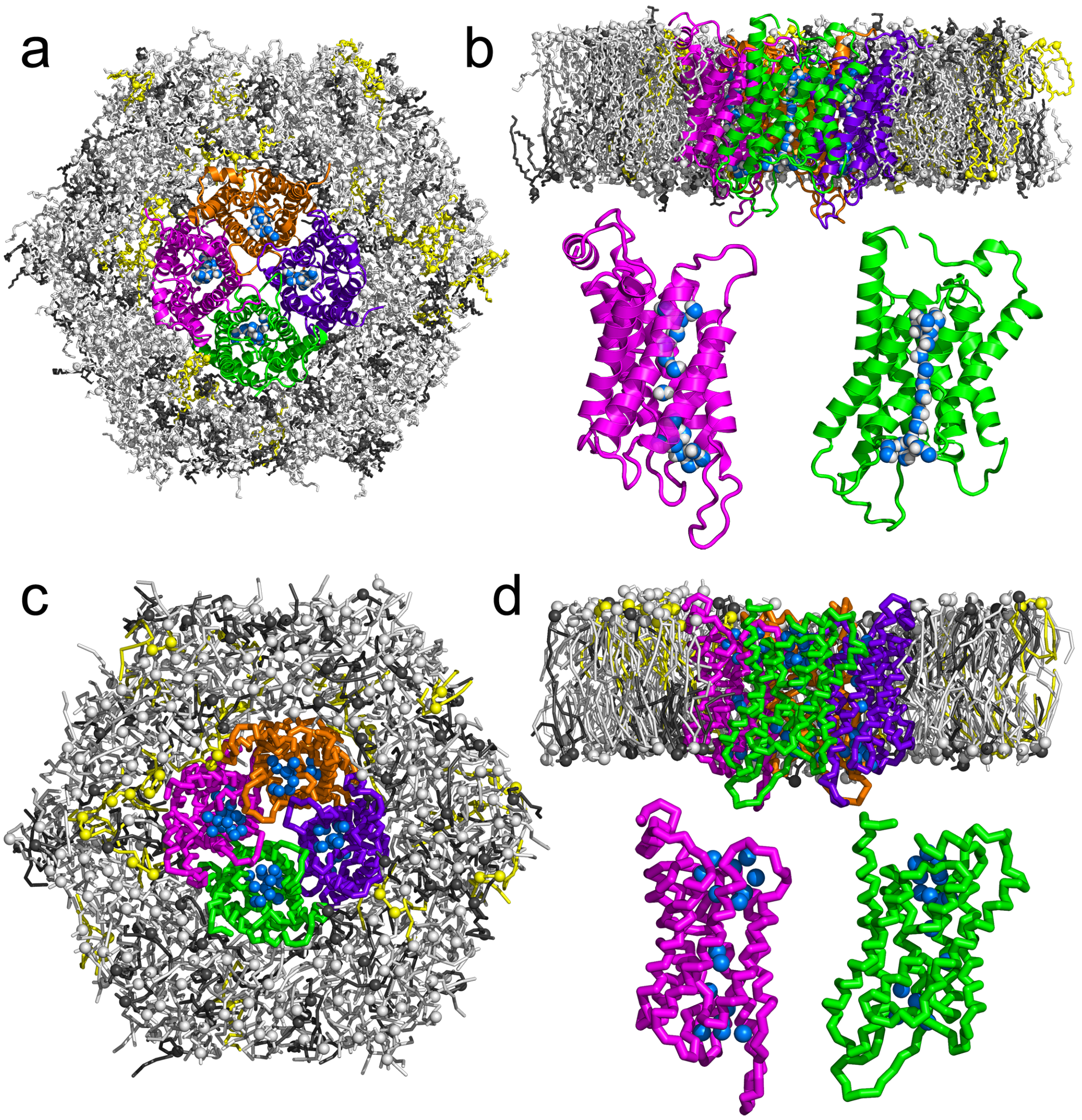

Figure S20: AQP1 embedded in the *E. coli* PLE *Avanti* model at 310 K at AA (a,b) and CG (c,d) resolution. Lipids are shown as sticks with phosphates highlighted as spheres. CLs are yellow, PGs are dark grey, PEs are light grey. AQP1 is shown as cartoon and ribbon in AA and CG, respectively, and colored chain-wise green, orange, magenta and purpleblue. The water molecules in the pore are colored in blue (with white hydrogens in AA resolution) and shown as spheres. Underneath the side views details of some individual chains with water in the pore are shown.

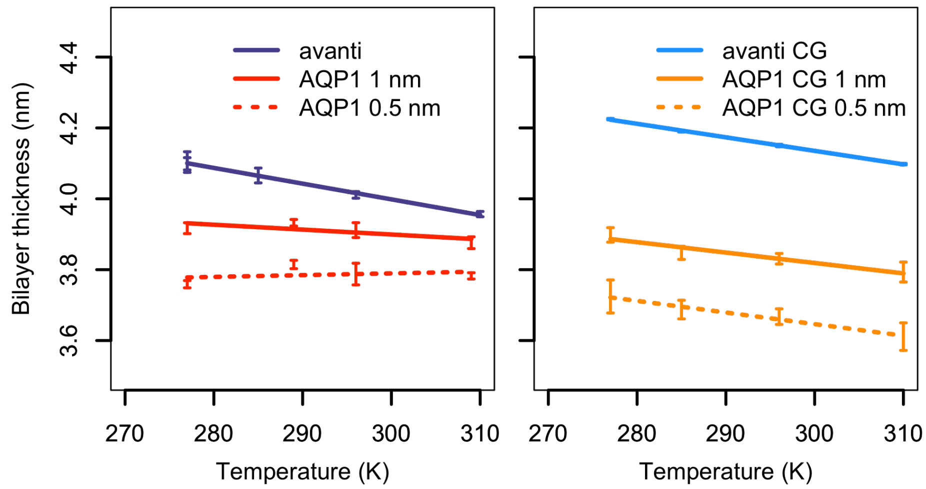

Figure S21: Phosphate-phosphate membrane thicknesses of *E. coli* PLE *Avanti* model in pure lipid systems and in 0.5 nm and 1 nm surroundings of AQP1 at different temperatures and at AA (left) and CG (right) resolutions.

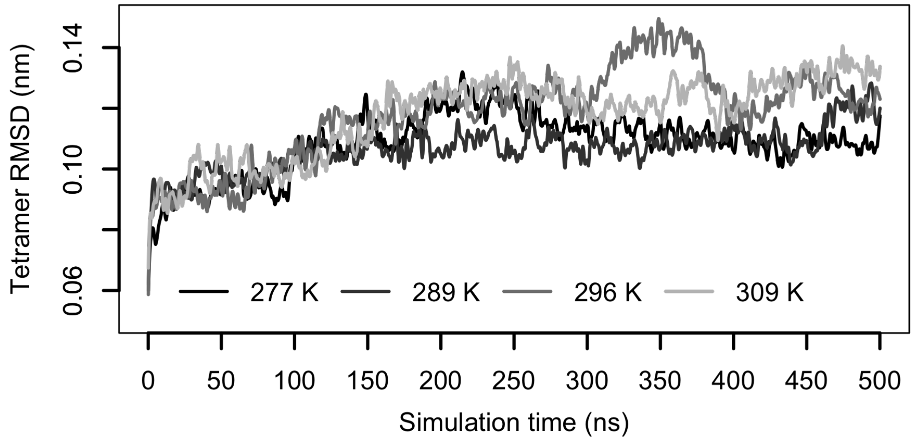

Figure S22: RMSD over time of C_α atoms within helices of the AA tetramer documenting the stability of the protein's quaternary structure in AA simulations.

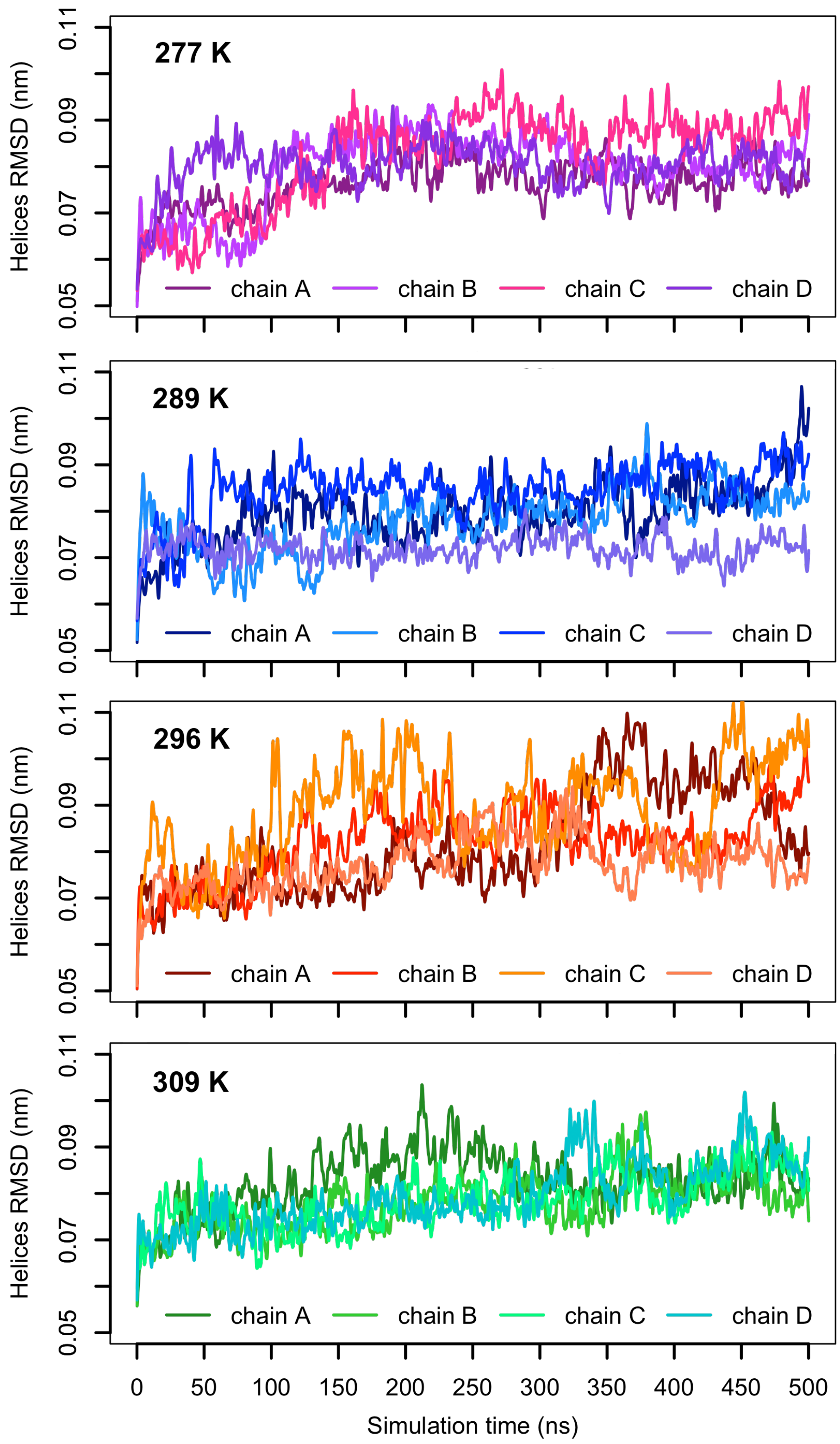

Figure S23: RMSD over time of C_α atoms within helices of the AA monomers documenting the stability of the protein's tertiary structure in AA simulations.

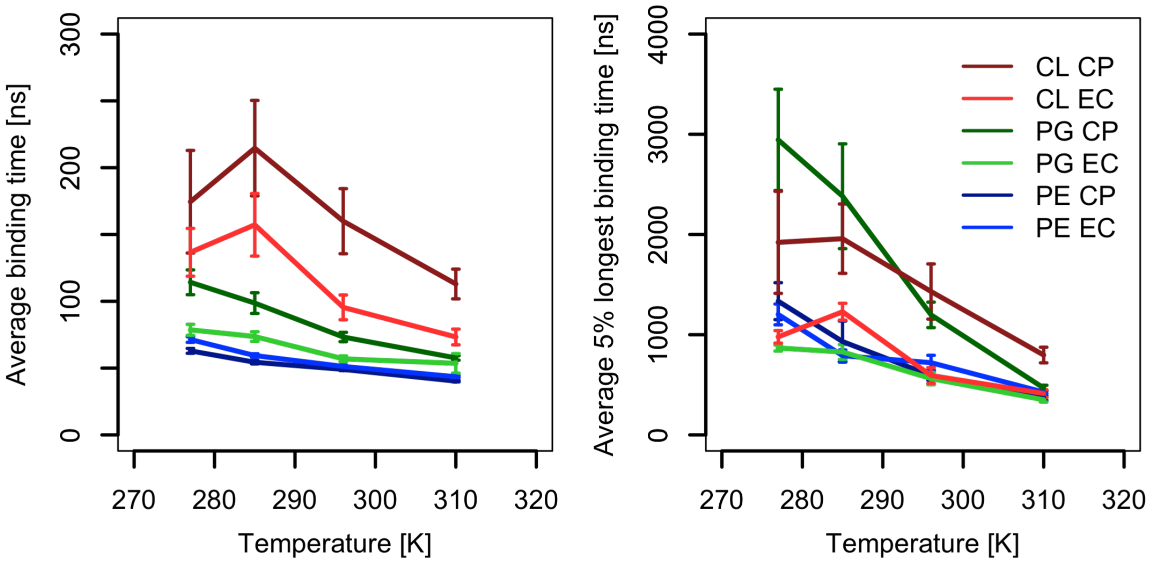

Figure S24: Lipid binding times to CG AQP1 at different temperatures. **Left:** Average binding times per lipid and layer of binding events longer than 10 ns. **Right:** Average binding times per lipid and layer of 5% longest binding events longer than 10 ns. CL denotes cardiolipin, PG phosphatidylglycerol, PE phosphatidylethanolamine, CP cytoplasmic layer, and EC extracellular layer. The error bars indicate standard errors of the mean. Binding events shorter than 10 ns were classified as random contacts and thus excluded from the analysis.

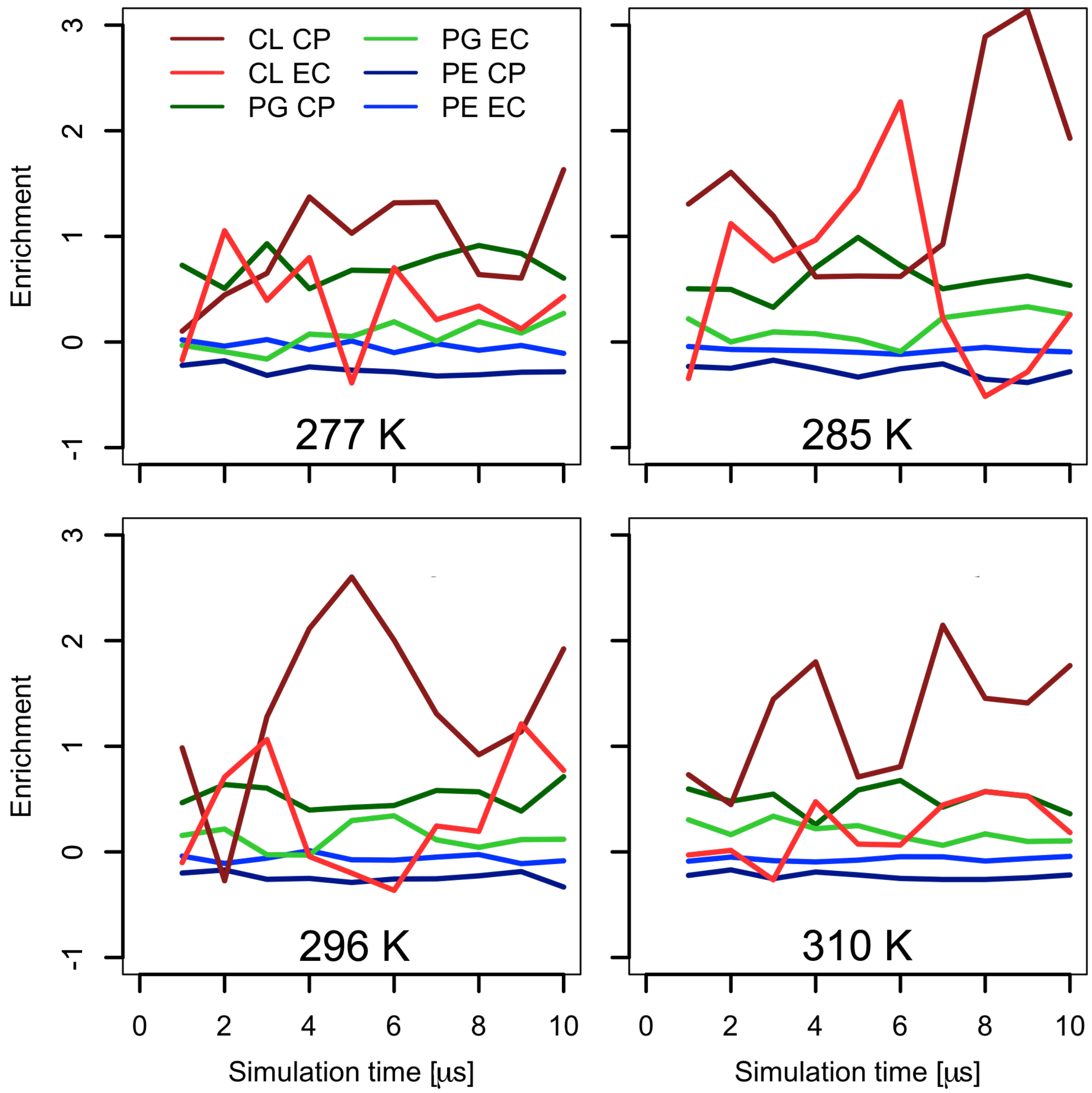

Figure S25: Time evolutions of lipid enrichment in the first solvation layer around CG AQP1. CL stands as a symbol for cardiolipin, PG for phosphatidylglycerol, PE for phosphatidylethanolamine, CP for cytoplasmic layer, and EC for extracellular layer. The error bars were excluded for clarity.
